# The Kondo Effect in Ce*_x_*LaLuScY (*x* = 0.05–1.0) High-Entropy Alloys

**DOI:** 10.3390/ma16247575

**Published:** 2023-12-09

**Authors:** Julia Petrović, Stanislav Vrtnik, Andreja Jelen, Primož Koželj, Jože Luzar, Peter Mihor, Qiang Hu, Magdalena Wencka, Bojan Ambrožič, Anton Meden, Goran Dražić, Sheng Guo, Janez Dolinšek

**Affiliations:** 1 Jožef Stefan Institute, Jamova 39, SI-1000 Ljubljana, Slovenia; 2 Faculty of Mathematics and Physics, University of Ljubljana, Jadranska 19, SI-1000 Ljubljana, Slovenia; 3 Institute of Applied Physics, Jiangxi Academy of Sciences, Changdong Road 7777, Nanchang 330096, China; 4 Institute of Molecular Physics, Polish Academy of Sciences, Smoluchowskiego 17, 60-179 Poznań, Poland; 5 Center of Excellence in Nanoscience and Nanotechnology, Jamova 39, SI-1000 Ljubljana, Slovenia; 6 Faculty of Chemistry and Chemical Technology, University of Ljubljana, Večna pot 113, SI-1000 Ljubljana, Slovenia; 7 Department of Materials Chemistry, National Institute of Chemistry, Hajdrihova 19, SI-1000 Ljubljana, Slovenia; 8 Industrial and Materials Science, Chalmers University of Technology, 41296 Göteborg, Sweden

**Keywords:** high-entropy alloys, structure and microstructure, Kondo effect

## Abstract

In the search for electronic phenomena in high-entropy alloys (HEAs) that go beyond the independent-electron description, we have synthesized a series of hexagonal rare earth (RE)-based HEAs: Ce*_x_*LaLuScY (*x* = 0.05–1.0). The measurements of electrical resistivity, magnetic susceptibility and specific heat have shown that the Ce*_x_*LaLuScY HEAs exhibit the Kondo effect, which is of a single impurity type in the entire range of employed Ce concentrations despite the alloys being classified as dense (concentrated) Kondo systems. A comparison to other known dense Kondo systems has revealed that the Kondo effect in the Ce*_x_*LaLuScY HEAs behaves quite differently from the chemically ordered Kondo lattices but quite similar to the RE-containing magnetic metallic glasses and randomly chemically disordered Kondo lattices of the chemical formula RE1*_x_*RE2_1−*x*_*M* (with RE1 being magnetic and RE2 being nonmagnetic). The main reason for the similarity between HEAs and the metallic glasses and chemically disordered Kondo lattices appears to be the absence of a periodic 4*f* sublattice in these systems, which prevents the formation of a coherent state between the 4*f*-scattering sites in the T→ 0 limit. The crystal-glass duality of HEAs does not bring conceptually new features to the Kondo effect that would not be already present in other disordered dense Kondo systems. This study broadens the classification of HEAs to correlated electron systems.

## 1. Introduction

Electronic phenomena in high-entropy alloys (HEAs) that emerge from electronic correlations, going beyond the independent electron description, remains as one of the unexplored topics. From the structural point of view, HEAs are conveniently divided into “first-generation” HEAs, denoting single-phase random solid solutions of five or more chemical elements in near equiatomic ratios on simple crystal lattices, like cubic close packed (ccp) (equivalent to face-centered cubic (fcc)), body-centered cubic (bcc) and hexagonal close packed (hcp), and “second-generation” HEAs, denoting multi-phase inhomogeneous mixtures of chemically disordered solid solutions and chemically ordered intermetallic compounds with either simple or complex crystal lattices (also termed compositionally complex alloys (CCAs)) [1,2]. Due to the simultaneous presence of a crystal lattice and an amorphous-type chemical (substitutional) disorder, HEAs show crystal-glass duality, sharing properties of both translationally periodic crystals and amorphous glasses. Because of this duality, an HEA material can be conveniently denoted as a *metallic glass on a crystal lattice*. For some single-phase solid solutions with statistically random distributions of chemical elements on the sites of a topologically weakly distorted lattice, a compositionally averaged unit cell or a supercell can be constructed, and the electronic properties can be treated within the nearly free electron (NFE) model [3]. This model considers the conduction electrons as weakly coupled to the ions of the crystal lattice, while the interactions between the electrons are neglected (i.e., the electrons are treated as independent). The NFE picture enables calculations of the electronic band structure, total energy and elastic constants of simple (usually first-generation) HEAs in the framework of density functional theory (DFT) on small-sized supercells [4,5,6], exact muffin tin orbital theory (EMTO) with coherent potential approximations (CPA) and effective medium theory [7,8].

There exists a class of metallic materials designated as correlated electron systems, which show interesting electronic phenomena that cannot be explained within the independent electron picture. An example is the Kondo problem of magnetic impurities in a nonmagnetic metallic host, representing quantum many-body physics well beyond the NFE description [9]. The Kondo effect is experimentally manifested as a minimum in the electrical resistivity with a −lnT dependence just below the minimum due to scattering of conduction electrons by the localized magnetic impurities, while in the T→ 0 limit, the magnetic moments of the impurities are screened by oppositely polarized spins of the conduction electrons through the formation of a collective Kondo singlet state with zero spin. It is tempting to consider whether the Kondo effect or other correlated electron phenomena also appear in HEAs and if (how) are they affected by their crystal-glass dual nature.

In this work, we report on the Kondo effect in a series of hexagonal rare earth (RE)-based HEAs with the nominal formula Ce*_x_*LaLuScY and nominal cerium contents *x* = 0.05, 0.1, 0.2, 0.5, and 1.0. The elements were selected based on their excellent mixing in a solid state and the fact that only Ce possesses a magnetic moment, while the other four elements are nonmagnetic. In this way, we obtain a clear situation of an HEA with close to randomly dispersed magnetic impurities in a nonmagnetic metallic host.

## 2. Materials Design, Synthesis, and Characterization

The target HEA material to search for the Kondo effect was a single-phase random solid solution of five chemical elements on a structurally simple crystal lattice, with one element being magnetic and the other four being nonmagnetic. The goal was to produce a series of metallic alloys in which the portion of the magnetic element would vary between low and abundant concentrations, including near-equiatomic HEA compositions. RE elements were chosen because of the great similarity in their chemical properties, which allowed for almost complete mutual solubility in a solid state. Out of 17 RE elements (15 lanthanides plus scandium and yttrium), only 4 are nonmagnetic (La, Lu, Sc, and Y) in a 3+ ionized state, and these were consequently employed. Cerium was chosen as the magnetic element because its binary mixing enthalpies with the four nonmagnetic elements are quite small. Ce is also exceptional among the RE elements because, being at the beginning of the lanthanide series (having one 4*f* electron), the spatial extent of its 4*f* wave function is the largest, and thus the exchange interaction between the 4*f* electron and the conduction electrons is the strongest. Ce and Yb are also the two RE elements which most frequently show the Kondo effect when bound in an intermetallic compound.

The binary mixing enthalpies $\Delta H_{mix}^{ij}$ of the five selected elements (Ce, La, Lu, Sc, and Y) are presented in Table 1, revealing that they are extremely close to zero (between 0 and 2 kJmol^−1^ [10]) for all elemental pairs *i* and *j*. This is a favorable situation for random mixing of the elements. Differences in the atomic radii, room-temperature (RT) crystal structures of the pure metals, ionic valences, and electronegativites are other important criteria for good solid solubility of the elements. According to the Hume-Rothery rules [11], single-phase random solid solutions are formed for minimal differences in the atomic radii, equal RT structures, similar valences, and similar electronegativities. Assuming the most probable 3+ valence state for all five elements, the last two criteria are well satisfied, while there are some concerns regarding the first two criteria (Table 2).

The atomic radii of Ce, La, and Y are quite similar (in the range of 0.182–0.188 nm), whereas the radius of Lu is a bit smaller (0.173 nm) while the radius of Sc is significantly smaller (0.165 nm) [10]. The largest radius difference is between La and Sc, amounting to 12%, which is large enough to introduce significant lattice deformation (strain) energy. In order to reduce the strain energy, random mixing of the elements may be compromised, and clustering of the smaller elements Sc and Lu into Sc,Lu-enriched domains and the larger elements Ce, La, and Y into Ce,La,Y-enriched domains may be preferred. Regarding the similarity of the RT crystal structures, the situation is more favorable. Lu, Sc, and Y crystallize in an hcp structure, whereas La crystallizes in a double hexagonal close-packed (dhcp) structure, which is quite similar to the hcp structure (the *c* lattice parameter of the dhcp structure is twice that of the hcp structure) [13]. Ce has two stable modifications at RT: dhcp and ccp. There is thus a good chance that the Ce*_x_*LaLuScY alloys will crystallize in a hexagonal structure.

The feasibility of producing solid solution phases in the Ce*_x_*LaLuScY (*x* = 0.05–1.0) system was assessed theoretically via the commonly used empirical criteria to predict solid solution formation in multicomponent alloys [2,14,15]. The results are presented in the Supplementary Materials (Ch. S-I), revealing that the formation of solid solution phases is highly probable. The question whether the expected solid solution phases fulfill the conditions to be termed as HEAs was addressed via the criterion by Yeh [16] (Ch. S-I of the Supplementary Materials), showing that they conformed to the definition of a high-entropy alloy.

The alloys were prepared by the method of arc melting in an Ar atmosphere. The ingots were remelted several times and cooled down naturally so that the alloys were in an as-cast state. Five Ce*_x_*LaLuScY alloys with the nominal cerium contents *x* = 0.05, 0.1, 0.2, 0.5, and 1.0 were prepared in order to systematically vary the amount of Ce magnetic “impurities” in the nonmagnetic LaLuScY metallic matrix. In the following, we shall designate the alloys by their nominal Ce contents (i.e., the alloy Ce_0.05_LaLuScY will be designated as *x* = 0.05), although the compositions determined by energy dispersive X-ray spectroscopy (EDS), which will be presented in the following, departed slightly from the nominal values. For the alloy with the equiatomic composition (and at the same time with the highest Ce content) *x* = 1.0, two ingots of the same nominal composition were prepared. One sample for measurements was cut from each ingot (the two samples are designated as *x* = 1.0a and *x* = 1.0b), and the same kind of experiments were performed on both. Doubling the number of samples for the *x* = 1.0 alloy is convenient for seeing the experimental error and reproducibility of the results obtained on two pieces of nominally the same alloy. Our investigations thus included six samples from five different Ce*_x_*LaLuScY alloys. All samples were in a polygrain morphology. The samples for measurements were cut from the inner parts of the ingots.

**Figure 1:**
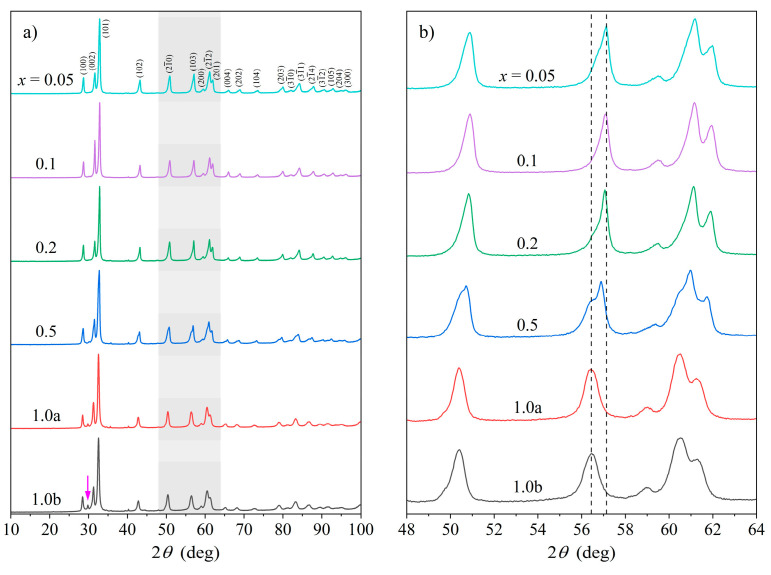
(**a**) XRD patterns of the Ce*_x_*LaLuScY (*x* = 0.05–1.0) HEAs at room temperature, with the Ce content *x* increasing from top to bottom. Miller indices *hkl* of the hcp-phase reflections are indicated in the *x* = 0.05 pattern. The arrow in the *x* = 1.0b pattern marks the most intense peak (111) of the ccp minority phase (at 2θ≈ 30^o^). (**b**) Zoomed-in parts of the XRD patterns in the range of diffraction angles 48° < 2θ < 64° (shaded light gray in panel (**a**)). The evolution of the doublet of peaks at 2θ= 56–57° with the cerium content is marked by the dashed vertical guidelines.

The crystal structures were identified by powder X-ray diffraction (XRD) using monochromatic Cu Kα_1_ radiation. The XRD patterns of all six samples in the entire investigated range of diffraction angles 10° < 2θ < 100°, with the Ce content increasing from top to bottom, are shown in Figure 1a. At first sight, the same phase (later shown to be hcp) dominated in all alloys, while more careful inspection of the XRD peaks reveals specific details that changed systematically with the increasing Ce content. This is demonstrated in the zoomed-in parts of the XRD patterns (48° < 2θ < 64°) shown in Figure 1b (also shaded light gray in Figure 1a) and is especially pronounced for the peak at 2θ= 56–57°. For the three lowest Ce contents *x* = 0.05, 0.1, and 0.2, the peaks were asymmetric, being less steep on the left (low-angle) side and steeper on the right (high-angle) side. This is an indication that there were in fact two phases present in the alloys, with both being of the same structural type but with slightly different sizes for the unit cells so that the reflections of the two phases overlapped. The less steep (left) side of the peak corresponds to the phase with a larger unit cell (denoted as L), while the high peak on the right corresponds to the phase with a smaller unit cell (S).

For the *x* = 0.5 sample, the difference in size of the two unit cells (and possibly the fractions of the two phases) was already large enough that a well-pronounced shoulder appeared on the left side of the main peak. For the two samples with the highest Ce content, *x* = 1.0a and *x* = 1.0b, only the phase with the larger unit cell was observable, and the peaks consequently became symmetric. As expected, the XRD patterns of these two samples were identical because they referred to the same alloy (*x* = 1.0) that was synthesized twice. The evolution of the doublet of peaks at 2θ= 56–57° with the cerium content is also marked by the dashed vertical guidelines in Figure 1b. The three samples with high Ce content *x* = 0.5, 1.0a and 1.0b additionally show the presence of a small amount of a third phase (later determined to be ccp). The most intense peak of this phase (at 2θ≈ 30°) is marked by a vertical arrow in the *x* = 1.0b pattern of Figure 1a.

**Figure 2:**
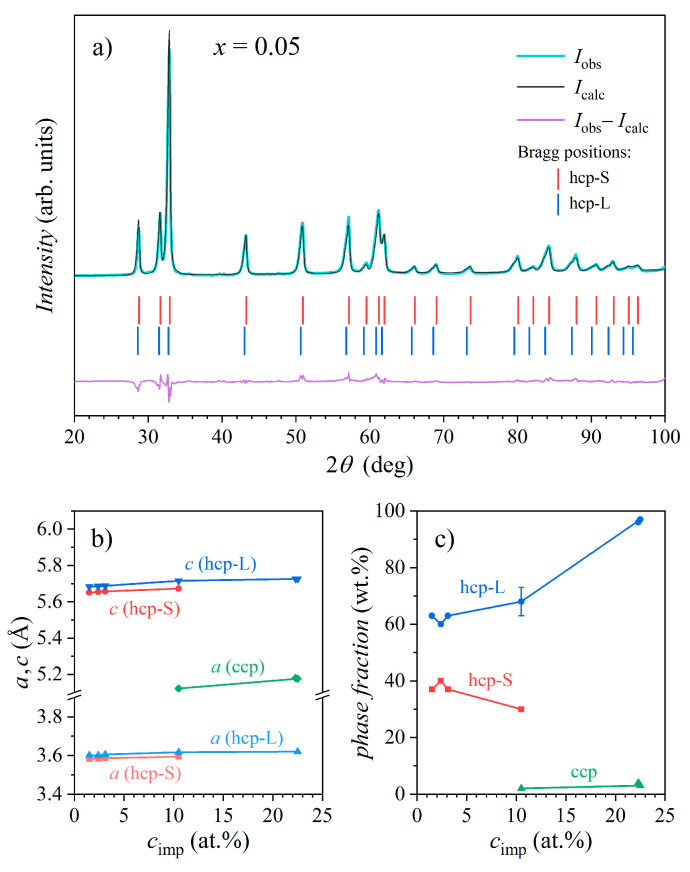
(**a**) XRD pattern of the *x* = 0.05 sample with the Rietveld refinement. The upper trace shows the observed intensity $I_{obs}$ (green) and the calculated intensity $I_{calc}$ (black) overlaid, whereas the bottom trace (purple) shows the difference ($I_{obs}-I_{calc}$). Bragg positions of the two hcp phases (hcp-S and hcp-L) are shown as stick spectra. (**b**) Lattice parameters and (**c**) phase fractions of the hcp-S, hcp-L, and ccp phases in the Ce*_x_*LaLuScY samples as a function of the Ce concentration $c_{imp}$ (solid lines connect the points).

To confirm the number of phases and quantify each phase crystallographically, Rietveld analysis using Topas Academic software Ver. 6 [17] was performed for all six samples. In the first step, Rietveld refinement was carried out for the *x* = 0.05 sample by considering only one hcp phase (space group *P*6_3_/*mmc*, No. 194). This reproduced all peak positions (with no peaks left unindexed), but the peak shapes did not fit well (the weighted profile residual factor $R_{wp}=$ 12.794% was quite large). In the next step, two hcp phases with slightly different sizes for the unit cells were assumed, and the shape and width of the peaks of each phase were fitted independently. This resulted in a significantly smaller value of $R_{wp}=$ 8.402%, which is convincing proof of the presence of two hcp phases. The Rietveld refinement of the *x* = 0.05 XRD pattern is shown in Figure 2a (Rietveld refinements for the alloys with other Ce contents *x* are not shown). The unit cell parameters of the hcp-S phase were a= 3.582 Å and c= 5.650 Å, while those of the hcp-L phase were a= 3.601 Å and c= 5.683 Å. The peaks of the hcp-L phase are significantly broader (signifying smaller or more disordered or distorted crystalline domains) than those of the hcp-S phase. The integrated intensities of the peaks yielded mass fractions of the two phases of $f_{hcp-S}=$ 37 wt.% and $f_{hcp-L}=$ 63 wt.%. (Note that although the peaks of the hcp-L phase are lower in amplitude than those of the hcp-S phase, they are broader, and thus the area under the peaks is larger such that, consequently, $f_{hcp-L}>f_{hcp-S}$.) The above crystallographic parameters, as well as those of all other samples, are collected in Table 3.

The same Rietveld refinement with two hcp phases (hcp-S and hcp-L) was also performed for the samples *x* = 0.1 and 0.2, again reproducing all peaks in the patterns well. For the *x* = 0.5 sample, a ccp minority phase (space group *Fm*$\bar{3}$*m*, No. 225) had to be added to the hcp-S and hcp-L majority phases. The lattice parameters of the three phases were a= 3.594 Å, c= 5.672 Å for the hcp-S phase, a= 3.617 Å and c= 5.715 Å for the hcp-L phase, and a= 5.122 Å for the ccp phase, while the phase fractions were $f_{hcp-S}=$ 30 wt.%, $f_{hcp-L}=$ 68 wt.%, and $f_{ccp}=$ 2 wt.%. For the two samples with the highest Ce content, *x* = 1.0a and 1.0b, the two hcp phases merged into a single one, which could be according to the value of the lattice parameters (a= 3.620 Å and c= 5.726 Å for both samples) and peak positions on the 2θ axis classified as the hcp-L phase. Both samples also contained the minority ccp phase of the fractions $f_{ccp}=$ 3 and 4 wt.% for *x* = 1.0a and 1.0b, respectively. By disregarding the ccp inclusions in the samples with high *x* values, the samples *x* = 0.05, *x* = 0.1, *x* = 0.2, and *x* = 0.5 are two-phase materials consisting of two identical hcp phases with a tiny difference in the unit cell size, while the two samples with equiatomic compositions *x* = 1.0a and *x* = 1.0b are single-phase hcp materials.

Considering that the experimental accuracy in the lattice parameter determination was about ±1 or ±2 on the third decimal, the lattice parameters from Table 3 show an increasing trend with the increasing Ce content in the alloys. This is demonstrated in Figure 2b, where these parameters are plotted as a function of the Ce concentration in the samples that was determined experimentally by the scanning electron microscopy (SEM) EDS method and conveniently denoted as the concentration of magnetic “impurities” $c_{imp}$, since Ce was the only magnetic element in the alloys. The phase fraction of the hcp-L phase also increased with increasing $c_{imp}$ values at the expense of the hcp-S phase (Figure 2c). The reason for this trend is straightforward: By introducing more Ce into the Ce*_x_*LaLuScY alloys, the element with a large atomic radius was added, replacing in part the elements with smaller atomic radii (Sc and Lu). Cerium was obviously incorporated into both hcp phases, which increased their lattice parameters with increasing $c_{imp}$ values.

By comparing the lattice parameter of the ccp phase (its average value over the three samples *x* = 0.5, 1.0a, and 1.0b amounted to $\bar{a}=$ 5.159 Å) to the literature data on the pure Ce ccp metal (a= 5.143 Å; see also Table 2) [12], it is reasonable to assume that Ce in the Ce*_x_*LaLuScY alloys at larger Ce contents started to build small cubic Ce-rich grains, despite the fact that most cerium was still dissolved in the two hcp phases. The fact that the a parameter of the cubic grains slightly increased with $c_{imp}$ (Figure 2b) indicates that the cubic grains were not pure Ce but also contained other (smaller) elements present in the alloys.

A comparison of the unit cell parameters of the hcp-S and hcp-L phases to the unit cell parameters of pure metals (Table 2) [12] indicates that the hcp-S phase was likely enriched in the two smaller elements Sc and Lu (or only Sc) but also contained other elements in similar concentrations, while the hcp-L phase was Sc- or Sc,Lu-deficient.

**Figure 3:**
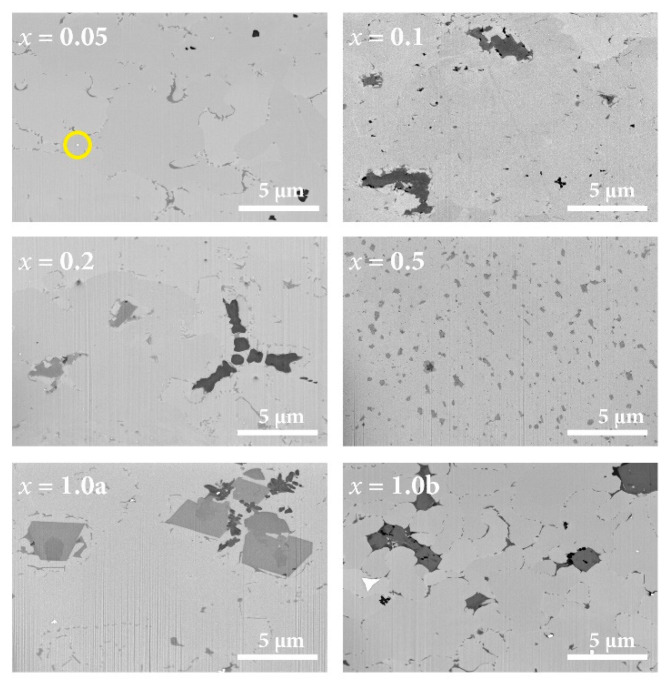
SEM BSE images of the Ce*_x_*LaLuScY (*x* = 0.05–1.0) samples. The yellow-circled tiny white spot in the image of the *x* = 0.05 sample represents tungsten carbide (WC) contamination of the material.

The microstructure of the alloys was examined via SEM backscattered electron (BSE) imaging. Imaging surfaces were prepared using the focused ion beam (FIB) technique using gallium ions. Micrographs of the six samples are shown in Figure 3. The microstructure was similar in all cases, showing crystalline grains of about a 10 µm cross-dimension, shaded slightly differently due to different crystallographic orientations (channeling contrast) and with some porosity and dark inclusions of a typical size (1 µm). EDS identified the dark inclusions (of various dark shades) to be, in most cases, yttrium oxide, while in some cases, the inclusions were strongly enriched in the lightest element (Sc). Some tiny white spots of about a 100 nm dimension were also visible (one of them is circled in yellow in the SEM BSE image of the *x* = 0.05 sample), representing tungsten carbide (WC) extrinsic contamination of the materials during preparation. Apart from the channeling contrast, the matrix appears to have a more or less uniform gray color. Except for the *x* = 1.0a and *x* = 1.0b samples, which were single-phase hcp materials (disregarding the ccp inclusions), the matrix of the other samples was the sum of the hcp-S and hcp-L phases, but the domains of the two phases could not be resolved by SEM BSE imaging due to their chemical compositions being too similar.

The chemical compositions were determined by EDS point analysis, whereas the spatial distributions of the chemical elements were examined by EDS elemental mapping. The matrix compositions of all samples (in at.%) are summarized in Table 3. Here, it is important to stress that for the two-phase samples, these compositions (denoted as SEM-EDS compositions) were averaged over the hcp-S and hcp-L phases due to the several µm size of the SEM interaction volume that was comparable to the size of the crystalline domains of the two phases. Individual compositions of the hcp-S and hcp-L phases were determined by the much more localized method of transmission electron microscopy (TEM) EDS, which will be presented later. The SEM-EDS compositions determined by point analysis (averaged over 10 randomly spaced points in the matrix) deviated slightly from the nominal compositions. In relation to the investigated Kondo effect, the most important concentration was the Ce concentration $c_{imp}$, where the nominal Ce concentrations in the Ce*_x_*LaLuScY samples *x* = 0.05, 0.1, 0.2, 0.5, 1.0a, and 1.0b recalculated in at.% were 1.2, 2.4, 4.8, 11.2, 20.0, and 20.0, respectively, while the experimental SEM-EDS concentrations were 1.5, 2.4, 3.1, 10.5, 22.5, and 22.3 (also given in a separate column in Table 3) and hence quite close to the nominal ones. This experimental set of $c_{imp}$ values will be used in the following for presenting various physical parameters as a function of the Ce concentration in the samples, while the SEM-EDS compositions from Table 3 will be used when calculating the magnetic and specific heat parameters per mole of the materials. Regarding the chemical composition of the yttrium-oxide inclusions, it is important to note that the oxide phase was nonmagnetic and electrically insulating, having no direct influence on the Kondo effect. For that reason, no effort was made to quantify their exact chemical composition.

**Figure 4:**
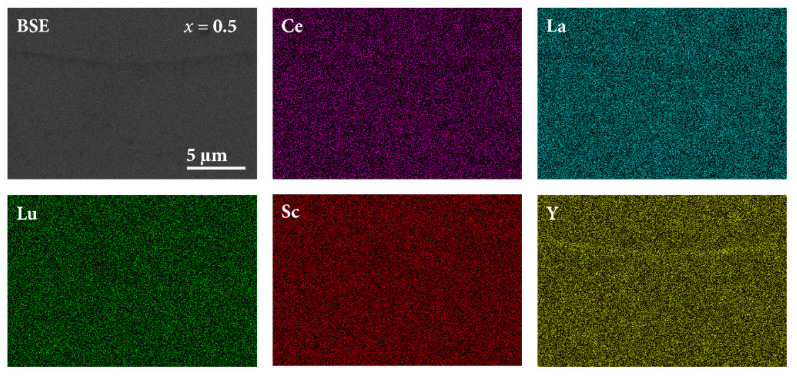
SEM BSE image (upper left panel) and SEM-EDS elemental maps of the *x* = 0.5 sample.

The SEM-EDS elemental maps of the *x* = 0.5 sample are shown in Figure 4, whereas the elemental maps of the other samples are given in Figures S1–S5 of the Supplementary Materials (Ch. S-II). A quite homogeneous dispersion of the five RE elements on the µm scale within the matrix is visible, with the compositional differences of the hcp-S and hcp-L phases being too small to be detected with this technique. The yttrium-oxide inclusions, as well as the Sc-rich inclusions depleted in Ce, La, and Y (the elements with large atomic radii, compared with the small Sc), both of a typical dimension of 1 µm, are also visible. The yttrium-oxide inclusions are best demonstrated in the elemental maps of the *x* = 1.0a sample (Figure S4 of the Supplementary Materials), where the oxygen map is also included. According to the SEM-EDS elemental maps, all investigated samples were close-to-random solid solutions of the five constituent chemical elements.

**Figure 5:**
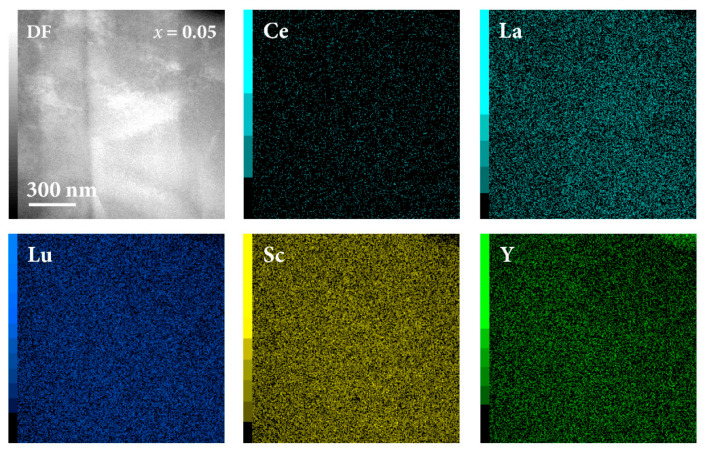
STEM-EDS elemental maps of the *x* = 0.05 sample. The STEM-DF image of the investigated area is shown in the upper left panel.

The nanostructure and the local chemical compositions on the nm scale were examined by scanning transmission electron microscopy (STEM) imaging and EDS elemental mapping using lamellae about 50 nm thick, which were prepared by FIB. The STEM dark-field (DF) image of the *x* = 0.05 sample is shown in the upper left corner of Figure 5, representing a 1.3 × 1.3 µm^2^ area on the surface where no defects were present. Other panels of Figure 5 show the elemental maps, where quite homogeneous distributions are evident, confirming random mixing of the five elements on the nanometric scale as well. The STEM-EDS composition obtained by point analysis (averaged over three points within the investigated area) was Ce_0.6_La_31.0_Lu_25.4_Sc_23.5_Y_19.5_, which is in reasonable agreement with the SEM-EDS composition Ce_1.5_La_31.0_Lu_31.0_Sc_17.1_Y_19.4_ from Table 3. The *x* = 0.05 sample is a two-phase system (consisting of the hcp-S and hcp-L phases), but it was not possible to assign the above STEM-EDS composition unambiguously to one of these phases because the second phase could not be found within the examined lamella. Investigations of the defects in other parts of the lamella yielded approximate compositions of the Sc-rich defects to be in the range from Sc_85_Lu_15_ to Sc_80_Lu_20_ (minor amounts of Y of less than 3 at.% were also detected). The defects were composed of the two small elements Sc and Lu, where the reason for this type of clustering has already been discussed (reduction in the lattice strain energy). Since the Sc,Lu-enriched defects were nonmagnetic (and small), they played no significant role in the Kondo effect.

The STEM-EDS investigations of the *x* = 0.2 sample are shown in Figure S6 of the Supplementary Materials (Ch. S-III), where the STEM-DF image and the EDS elemental maps of the area that contained both the hcp-S and hcp-L phases, the Sc,Lu-rich defects, the yttrium-oxide regions, and the tungsten carbide (WC) contamination are shown. The results are in agreement with those of the *x* = 0.05 sample presented in Figure 5 and additionally give the STEM-EDS compositions of the individual hcp-S and hcp-L phases.

## 3. Results

### 3.1. Electrical Resistivity

**Figure 6:**
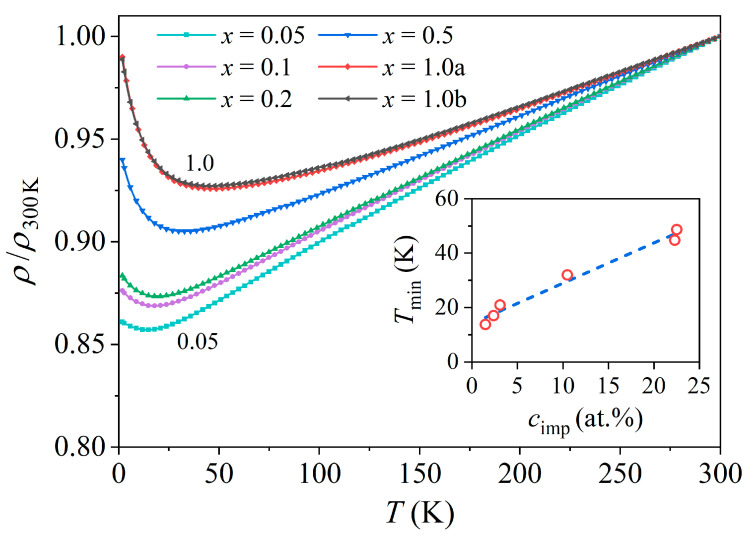
Temperature-dependent electrical resistivity of the Ce*_x_*LaLuScY (*x* = 0.05–1.0) samples in zero magnetic field. The resistivities are presented as normalized to their 300 K values, $\rho \left(T\right)/\rho_{300K}$. The inset shows the temperature of the resistivity minimum $T_{min}$ as a function of the Ce concentration $c_{imp}$ in the samples (dashed line is a guide for the eye).

The electrical resistivity of the six Ce*_x_*LaLuScY samples in the entire investigated temperature range of 0.35–300 K in a zero magnetic field (B= 0) is shown in Figure 6. The resistivities are presented as normalized to their 300 K values, $\rho \left(T\right)/\rho_{300K}$, while the raw data (not normalized) are shown in Figure S7 of the Supplementary Materials (Ch. S-IV). The raw data were affected by the experimental error that came from the uncertainty in determination of the samples’ length and cross section and the possible inhomogeneity of the electrical contacts. Due to this error, the absolute resistivity values of the six samples scattered randomly (uncorrelated with the Ce content *x*) by about ± 5% around the average value that amounted at RT to 150 µΩcm. When analyzing the normalized resistivity $\rho \left(T\right)/\rho_{300K}$, these errors were compensated for, and the dependence on the Ce content became evident.

Away from the low-temperature regime, the resistivities were of a simple metallic type, increasing linearly upon heating, while the positive temperature coefficient decreased systematically with an increasing Ce concentration. At low temperatures, the resistivities exhibited a minimum, and the temperature of the minimum $T_{min}$ shifted to higher temperatures linearly with an increasing Ce concentration $c_{imp}$ (from $T_{min}=$ 14 K for the *x* = 0.05 sample to $T_{min}=$ 45 ± 2 K for the *x* = 1.0a and 1.0b samples), as shown in the inset of Figure 6.

**Figure 7:**
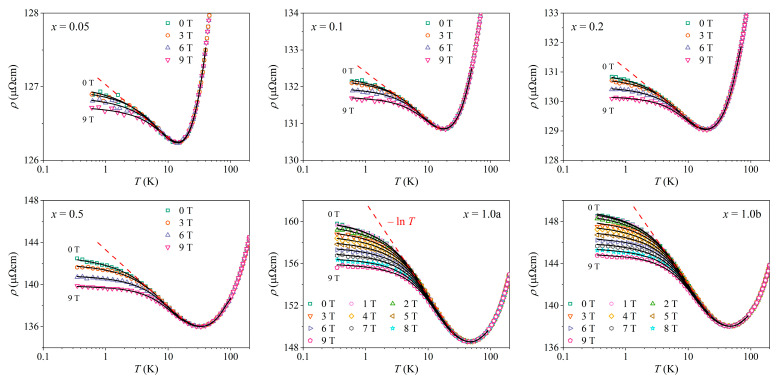
Temperature-dependent resistivity ρ in magnetic fields B= 0–9 T on a logarithmic temperature scale, shown in a separate panel for each sample. The red dashed lines indicate the ρ∝−lnT dependence below the resistivity minimum. Solid curves are fits with the Hamann formula in Equation (1), which is described in the text.

In Figure 7, the temperature-dependent resistivity ρ in magnetic fields B= 0–9 T is shown on a logarithmic temperature scale, with each sample in a separate panel. For the samples *x* = 0.05, 0.1, 0.2, and 0.5, the magnetic field was varied in steps of ΔB= 3 T, whereas for the samples *x* = 1.0a and 1.0b, the step was ΔB= 1 T. Below the minimum, there was first a pronounced temperature range where the resistivities show a ρ∝−lnT dependence (indicated by a dashed line), whereas in the T→ 0 limit, the resistivities show a tendency to level off toward a horizontal plateau. Such temperature dependence is characteristic of single-impurity type Kondo systems. The resistivities became magnetic field-dependent below the resistivity minimum. In an increasing magnetic field, the resistivities decreased, which is again characteristic of the Kondo effect. The mechanism of the Kondo resistivity is based on spin coupling between the impurity spin and the spins of conduction electrons, where thermally fluctuating impurity spins introduce spin-flip transitions of the conduction electrons. In an increasing external magnetic field, the impurity moments are progressively locked by the field, which reduces their fluctuations, and the probability of spin-flip transitions of conduction electrons consequently diminishes, resulting in a reduction in the resistivity. Figure 7 shows that the decrease was relatively small even in the highest applied field of 9 T, and the overall temperature dependence $\rho \left(T\right)$ remained qualitatively the same in all fields, indicating that the Kondo effect in the Ce*_x_*LaLuScY HEAs was robust against the magnetic field in the investigated range of 0–9 T.

The resistivity in the temperature regime of the Kondo effect (between the lowest investigated temperature of 0.35 K and several tens of Kelvin above the resistivity minimum) was analyzed by the theory of Hamann. The Hamann resistivity formula [18] using the theoretical approach by Nagaoka [19] is expressed as follows:

(1) $$\rho =\rho_{0}+qT^{2}+pT^{5}+\rho_{1}\left\{1-\frac{ln\left(T/T_{K}\right)}{{\left[{ln}^{2}\left(T/T_{K}\right)+S\left(S+1\right)\pi^{2}\right]}^{1/2}}\right\}.$$

The first three terms ($\rho_{0}+qT^{2}+pT^{5}$) describe the resistivity of a “normal” (nonmagnetic) metal, where $\rho_{0\;}$ is the residual (T= 0) resistivity due to static quenched disorder in the crystal lattice, $qT^{2}$ is the low-temperature expression for the resistivity due to electron–electron correlations (Coulomb repulsion), and $pT^{5}$ is the low-temperature expression for the electron-phonon coupling (the Bloch–Grüneisen law). The Kondo part of the resistivity is the term $\rho_{1}\left\{\ldots \right\}$, with

(2) $$\rho_{1}=2\pi c_{imp}/\left(n_{e}e^{2}k_{F}\right),$$

where $c_{imp}$ is the Ce concentration, $n_{e}$ is the electron density, $k_{F}$ the Fermi wave vector, $T_{K}$ the Kondo temperature, and S is the impurity spin. The Hamann formula was originally developed for spin S= 1/2 magnetic impurities, but it was later realized that the fitting routine could not converge for spin S≥ 1/2, and thus the formula was empirically modified to treat S as a free parameter [20,21,22], allowing it to also be used for higher spins. The final fit-determined values of the S parameter reported in the literature are usually in the range of 0.1–0.3. The fit parameters of the Kondo part of the resistivity are $\rho_{1}$, $T_{K}$, and S. Equation (1) reproduces analytically the electrical resistivity of a Kondo system in a broad temperature interval from far above the minimum down to the T→ 0 limit. (In contrast, the original theory by Kondo breaks down upon reaching below the Kondo temperature $T_{K}$.)

**Figure 8:**
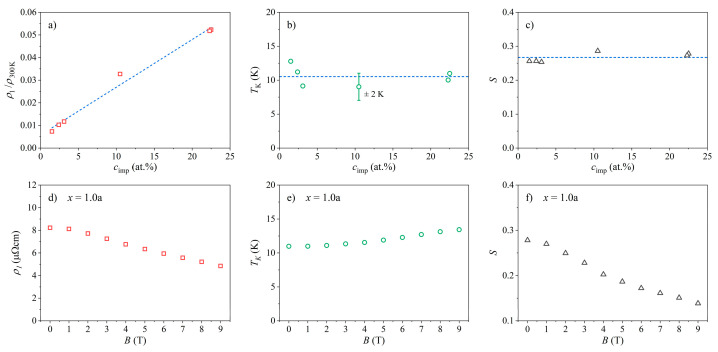
Dependence of the fit parameters related to the Kondo term in the Hamann resistivity formula of Equation (1) on the Ce concentration, determined from the zero-field resistivities: (**a**) $\rho_{1}$ vs. $c_{imp}$ (the $\rho_{1}$ values are normalized to $\rho_{300K}$), (**b**) $T_{K}$ vs. $c_{imp}$, and (**c**) S vs. $c_{imp}$. Dashed lines are guides for the eye. The dependence of the same fit parameters on the magnetic field B for the sample *x* = 1.0a: (**d**) $\rho_{1}$ vs. B, (**e**) $T_{K}$ vs. B, and (**f**) S vs. B.

The fits of the resistivity with Equation (1) in magnetic fields B= 0–9 T were performed for all six samples and all applied fields. The fits are shown as solid curves in Figure 7. We observed that the Hamann formula gave an excellent fit to the experimental data for all samples in a broad temperature range from the lowest temperature of 0.35 K up to the temperatures of several tens of Kelvin above the resistivity minimum. The behavior of the fit parameters related to the Kondo term in the resistivity ($\rho_{1}$, $T_{K}$, and S) as a function of the Ce concentration $c_{imp}$, determined from the zero-field resistivities, is shown in Figure 8. The $\rho_{1}\left(c_{imp}\right)$ relation is presented in Figure 8a. (To compensate for the above-described experimental errors, the normalized values $\rho_{1}/\rho_{300K}$ are shown.) Since $\rho_{1}$ of Equation (2) is directly proportional to the Ce concentration, a linear $\rho_{1}/\rho_{300K}$ vs. $c_{imp}$ relation was expected, which was indeed observed experimentally. The dependence of the Kondo temperature $T_{K}$ on the Ce concentration, $T_{K}\left(c_{imp}\right)$, is shown in Figure 8b. The $T_{K}$ values scattered randomly within the interval $T_{K}=$ 11 ± 2 K in the entire $c_{imp}$ range, and thus $T_{K}$ can be considered $c_{imp}$-independent within the precision of the Hamann fits. This result points toward the single-impurity character of the observed Kondo effect (each magnetic impurity scattered the conduction electrons independently). The relation between the impurity spin and the Ce concentration $S\left(c_{imp}\right)$ is presented in Figure 8c, where the effective spin values are in the interval S= 0.27 ± 0.02, also being $c_{imp}$-independent.

The Hamann formula itself does not predict any explicit field dependence of the Kondo resistivity, yet it also fits the resistivity data measured in a magnetic field well. In this situation, the dependence of the fit parameters $\rho_{1}$, $T_{K}$, and S on the magnetic field B has to be taken as the experimental fact. In Figure 8d–f, we show the $\rho_{1}\left(B\right)$, $T_{K}\left(B\right)$, and $S\left(B\right)$ relations, respectively, for the sample *x* = 1.0a, where the measurements were conducted with the field resolution ΔB= 1 T. The $\rho_{1}$ parameter slightly decreased with increasing B, $T_{K}$ showed a small increase from 11 K in a zero field to 12.5 K in a 9 T field, and S decreased monotonously from the value 0.28 in a zero field to 0.14 in a 9 T field. The $\rho_{1}\left(B\right)$, $T_{K}\left(B\right)$, and $S\left(B\right)$ relations for all six investigated samples are presented in Figure S8 of the Supplementary Materials (Ch. S-V).

### 3.2. Magnetic Susceptibility

The temperature-dependent magnetization $M\left(T\right)$ and magnetic susceptibility $\chi \left(T\right)$ were determined in the temperature range of 1.8–350 K in magnetic fields $\mu_{0}H$ between 0.1 and 7 T. Both zero-field-cooled (zfc) and field-cooled (fc) magnetization measurements were performed. Some samples contained tiny FM contamination of an extrinsic origin (introduced during material synthesis), while others were free of such contamination. The FM contamination manifested as hysteresis in the magnetization versus the magnetic field, $M\left(H\right)$, curves that persisted up to the highest investigated temperature of 350 K, and the hysteresis loops closed up in a field $\mu_{0}H\approx$ 0.3 T, which is a typical value for FM-type loops. In addition, the FM contamination also introduced zfc-fc splitting of the $M\left(T\right)$ curves observable in small magnetic fields up to the highest temperature. To demonstrate the FM contamination, the $M\left(H\right)$ curves of the *x* = 0.1 non-contaminated sample and the *x* = 0.5 FM-contaminated sample are compared in Figure S9 of the Supplementary Materials. In the following, we present the magnetic results for the *x* = 0.1 non-contaminated sample, which are representative of all six samples. (In addition, the *x* = 0.1 sample also did not contain any Ce-rich ccp precipitates that could aggravate the analysis.)

**Figure 9:**
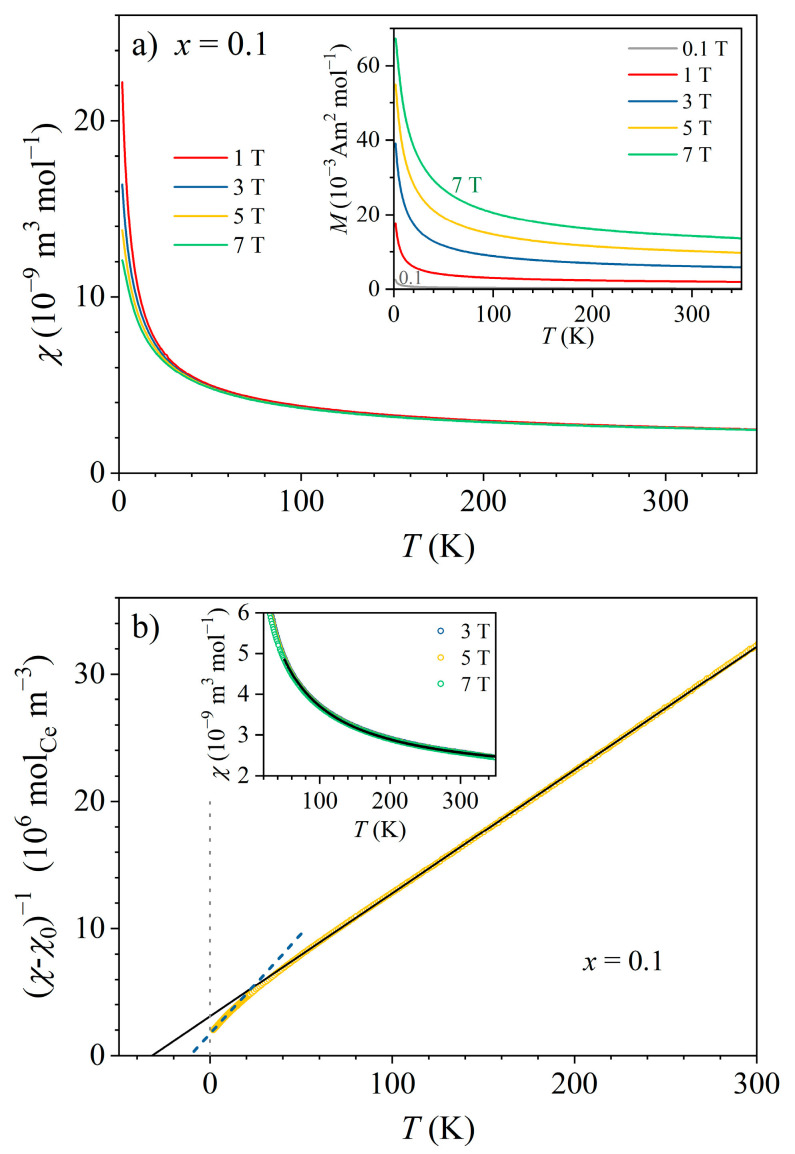
(**a**) Magnetic susceptibility χ=M/H of the *x* = 0.1 sample in the temperature range 1.8–350 K in different magnetic fields (indicated in the legend). The inset shows the temperature-dependent magnetization $M\left(T\right)$ curves. (**b**) ${\left(\chi -\chi_{0}\right)}^{-1}$ versus temperature for the magnetic field of 5 T. The solid line is the Curie–Weiss fit at temperatures above 50 K, while the dashed line is the Curie–Weiss fit in the low-temperature range 1.8–9 K. The inset shows the total susceptibility χ vs. T on an expanded vertical scale, together with the fit with Equation (3) (solid curve) in the temperature range 50–300 K (the experimental data in magnetic fields 3, 5, and 7 T are indistinguishable on the graph).

The $M\left(T\right)$ curves of the *x* = 0.1 sample in magnetic fields $\mu_{0}H=$ 0.1, 1, 3, 5, and 7 T are presented in the inset of Figure 9a. The curves show Curie–Weiss paramagnetic behavior, which is superimposed on a positive, temperature-independent “foot”. No difference between the zfc and fc magnetizations could be observed at any temperature in any magnetic field. The susceptibility χ=M/H is presented in the main panel of Figure 9a, where it is evident that the curves in all magnetic fields overlap in the temperature range of 50–350 K, confirming that the total magnetization depended linearly on the magnetic field. Below about 50 K, a weak field dependence can be noticed. The susceptibility for T> 50 K can be written as the sum of a temperature-independent term $\chi_{0}$ and a Curie-Weiss term $\chi_{CW}$:

(3) $$\chi =\chi_{0}+\chi_{CW}=\chi_{0}+C_{CW}/\left(T-\theta_{CW}\right).$$

The quantities appearing in the Curie–Weiss susceptibility are the Curie–Weiss temperature $\theta_{CW}$ and the Curie–Weiss constant $C_{CW}=\mu_{0}n_{\mu }\mu_{eff}^{2}/3k_{B}$, where $n_{\mu }$ denotes the number density of the magnetic moments, $\mu_{eff}$ is the effective magnetic moment of the magnetic ions (Ce^3+^ in this case), and $\mu_{0}$ is the induction constant [23]. The temperature-independent term $\chi_{0}$ is generally the sum of the positive Pauli spin susceptibility of conduction electrons, the negative Larmor diamagnetic susceptibility of the electronic cores, and the negative Landau diamagnetic susceptibility due to the conduction electron circulation in a magnetic field such that $\chi_{0}=\chi_{Pauli}+\chi_{Larmor}+\chi_{Landau}$. The fit of the total susceptibility with Equation (3) in the high-temperature range above 50 K is shown in the inset of Figure 9b, where good agreement with the experimental data is evident. The fit parameter values are $\chi_{0}=$ 1.8 × 10^−9^ m^3^mol^−1^ and $\theta_{CW}=$ −32 K, and the effective magnetic moment (extracted from $C_{CW}$) $\mu_{eff}=$ 2.56$\mu_{B}$, where $\mu_{B}$ is the Bohr magneton. The negative $\theta_{CW}$ value indicates the presence of AFM interactions between the Ce moments (of the indirect exchange Ruderman–Kittel–Kasuya–Yosida (RKKY) type), but the interactions were not strong enough to induce collective spin ordering, and the material remained paramagnetic down to the lowest investigated temperature of 1.8 K. The effective moment of 2.56$\mu_{B}$ is practically identical to the theoretical paramagnetic moment of a Ce^3+^ ion that amounts to 2.54$\mu_{B}$. The positive $\chi_{0}$ value indicates that its origin is predominantly Pauli spin susceptibility. This can be qualitatively tested by recalling that for free electrons, $\chi_{Pauli}$, $\left|\chi_{Larmor}\right|$, and $\left|\chi_{Landau}\right|$ are of the same order of magnitude. Calculating the Larmor susceptibility from the literature tables [24] yielded $\chi_{Larmor}=$ −0.175 × 10^−9^ m^3^mol^−1^, which is smaller (in the absolute sense) than the $\chi_{0}$ determined from the fit by a factor of 10, supporting the predominant Pauli origin of the latter. (In the following, $\chi_{0}$ will be conveniently denoted as the Pauli susceptibility.)

In the next step, $\chi_{0}$ was subtracted from the total susceptibility, and ${\left(\chi -\chi_{0}\right)}^{-1}$ was plotted versus the temperature (main panel of Figure 9b). The fit of the 5 T data with Equation (3) (a straight line in this kind of a plot) is also shown, confirming the Curie–Weiss behavior at temperatures above 50 K, with the $\theta_{CW}$ and $\mu_{eff}$ values determined before from the $\chi \left(T\right)$ fit. Below that temperature, the experimental data start to deviate from the high-temperature Curie–Weiss line in a downward curvature. In the low-temperature range between 1.8 and 9 K, another Curie–Weiss fit could be performed (dashed line in Figure 9b), yielding a smaller effective moment $\mu_{eff}=$ 2.01$\mu_{B}$ and smaller (absolute) Curie–Weiss temperature $\theta_{CW}=$ −11 K. This behavior can be interpreted by the Kondo effect, where below $T_{K}$ (11 K in this case, as determined from the electrical resistivity), the conduction electrons started to screen magnetically the Ce moments, resulting in a reduction of $\mu_{eff}$. The RKKY interaction between the adjacent Ce moments was then weakened, which manifested in the reduction in the (absolute) $\theta_{CW}$ value. However, the effect of a crystal electric field (CF) can also reduce the magnetic moment at low temperatures, and since the CF energy level scheme for the Ce*_x_*LaLuScY HEAs is not known yet, it is not possible to discriminate between these two effects and claim unambiguously the sole Kondo origin of the low-temperature deviation from the high-temperature Curie–Weiss behavior.

The magnetic data of the samples that contained the FM contamination were analyzed by the following subtraction procedure. The magnetization in this case assumes the form $M=M_{0}+M_{CW}+M_{FM}$, where $M_{0}=\chi_{0}H$ and $M_{CW}=\chi_{CW}H$, whereas $M_{FM}$ is the magnetization of the FM contamination. In a large enough external field, $M_{FM}$ is saturated (i.e., does not depend on the field anymore), and thus the subtraction of two magnetizations measured in different magnetic fields $H_{1}$ and $H_{2}$ (both large enough that $M_{FM}$ is already saturated) eliminates the FM contribution, yielding the “differential” magnetization $\Delta M=M\left(H_{2}\right)-M\left(H_{1}\right)=\left(\chi_{0}+\chi_{CW}\right)\left(H_{2}-H_{1}\right)$. The analysis of the susceptibility then proceeds along the same steps as for the samples without the FM contamination, but as demonstrated in Figure S10 of the Supplementary Materials (Ch. S-VI), the precision of the differential analysis was a bit lower at low temperatures, and the Curie–Weiss fit parameters of the FM-contaminated samples were less accurate, while the parameter $\chi_{0}$ was reliably determined.

**Figure 10:**
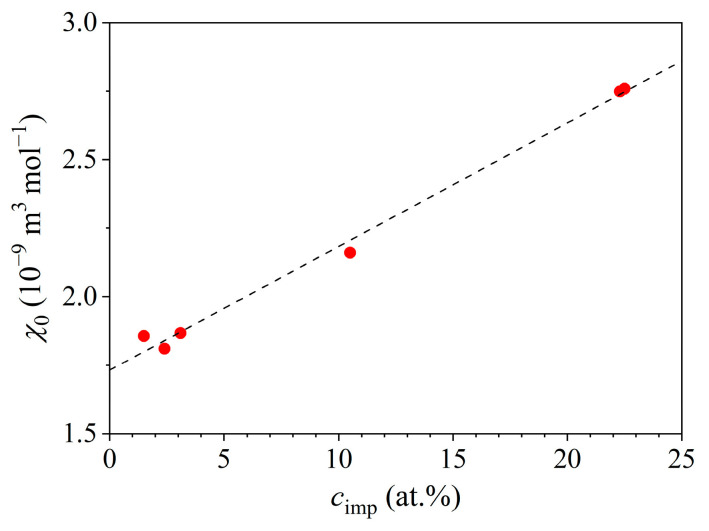
Pauli spin susceptibility $\chi_{0}$ of the investigated samples as a function of the Ce concentration $c_{imp}$. The dashed line is a linear fit.

The Pauli susceptibility $\chi_{0}$ was determined for all six samples, and it is shown as a function of the Ce concentration in Figure 10, where a linear increase in $\chi_{0}$ with an increasing $c_{imp}$ is evident. The meaning of this result will be discussed in the Section 4.

### 3.3. Specific Heat

The specific heat of a metallic alloy containing magnetic elements is a sum $C=C_{el}+C_{latt}+C_{mag}$, where $C_{el}$ is the electronic contribution, $C_{latt}$ the lattice (phononic) contribution, and $C_{mag}$ is the magnetic contribution to the total specific heat. The electronic contribution is linear in terms of temperature up to the melting point of the alloy $C_{el}=\gamma T$, where $\gamma =\left(\pi^{2}/3\right)k_{B}^{2}g\left(\varepsilon_{F}\right)$ is the electronic specific heat coefficient that is directly proportional to the electronic density of states (DOS) $g\left(\varepsilon_{F}\right)$ at the Fermi energy $\varepsilon_{F}$. The lattice contribution at low temperatures can be written in the Debye model as $C_{latt}=\alpha T^{3}$ with $\alpha =12\pi^{4}R/5\theta_{D}^{3}$, where $\theta_{D}$ is the Debye temperature and R is the gas constant. The magnetic contribution $C_{mag}$ represents the heat released from the material due to lowering of the exchange energy of the spin system by spin ordering. The ordering can either be collective like FM, AFM, or spin glass or of a single-ion type like the single-impurity Kondo effect and the Schottky effect. In addition, the low-temperature excitations (magnons) of the collectively ordered phases contribute to the magnetic specific heat at T→ 0 as well.

**Figure 11:**
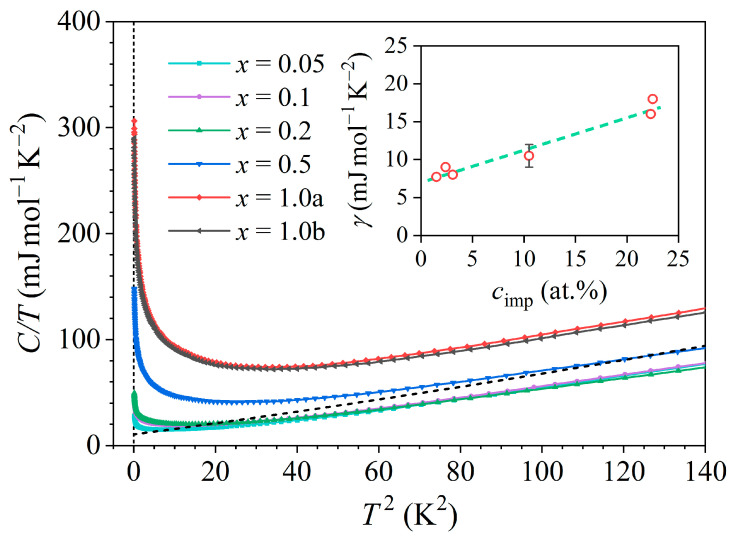
Zero-field specific heat of the six Ce*_x_*LaLuScY samples at temperatures below 12 K in a C/T vs. $T^{2}$ plot. Dashed line is a $\gamma +\alpha T^{2}$ fit for the *x* = 0.5 sample. The inset shows the electronic coefficient γ as a function of the cerium concentration $c_{imp}$.

Specific heat measurements of the six Ce*_x_*LaLuScY samples were performed in the temperature range 0.35–300 K in magnetic fields of 0–9 T. The low-temperature, zero-field (B= 0) specific heat of all six samples at temperatures below 12 K in a C/T versus $T^{2}$ plot is shown in Figure 11. In this kind of a plot, the electronic and lattice contributions $\left(C_{el}+C_{latt}\right)/T=\gamma +\alpha T^{2}$ yielded linear dependence with a slope α and zero-temperature intercept of the vertical axis γ. We observe that the total specific heat curves indeed show such a linear range at temperatures roughly above 8 K, while the upturn at lower temperatures can be attributed to the magnetic contribution. The fits $\gamma +\alpha T^{2}$ in the linear regime were made for all samples, but for clarity of presentation, only the fit for the *x* = 0.5 sample is shown as a black dashed line in Figure 11.

The coefficient γ increased linearly with the increasing cerium concentration $c_{imp}$ (inset in Figure 11), having the value γ= 7.7 mJmol^−1^K^−2^ for the *x* = 0.05 sample and going up to γ= 17 ± 1 mJmol^−1^K^−2^ for the *x* = 1.0a and *x* = 1.0b samples (recall that the γ values of the constituent pure metals were [25] $\gamma_{Ce}=$ 12.8, $\gamma_{La}=$ 9.45, $\gamma_{Lu}=$ 8.19, $\gamma_{Sc}=$ 10.34, and $\gamma_{Y}=$ 8.2 in the unit mJmol^−1^K^−2^). A remarkable observation in Figure 11 is that the C/T data of all samples in the linear regime are parallel, signaling that the lattice’s specific heat was the same for all samples. This is a plausible result due to the fact that all alloys share a common hexagonal crystal structure and the hexagonal unit cell parameters a and c changed very little with the cerium concentration (Figure 2b), which makes their phonon spectra quite similar. The Debye temperature $\theta_{D}$ extracted from the lattice’s specific heat coefficients α was about the same for all samples, amounting to $\theta_{D}=$ 140 ± 10 K. It is also important to note that the C/T data from Figure 11, which are presented per mole of the samples (i.e., per mole of the Ce*_x_*LaLuScY alloys), increased in magnitude systematically with an increasing Ce content *x*. 

**Figure 12:**
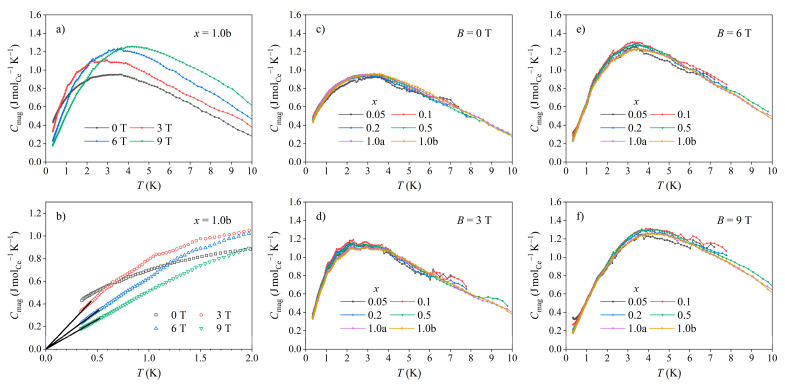
(**a**) Magnetic specific heat of the *x* = 1.0b sample in magnetic fields of 0, 3, 6 and 9 T in a $C_{mag}$ vs. T plot. (**b**) The same data on an expanded temperature scale in the T→ 0 limit to demonstrate linear vanishing of the 3 T, 6 T, and 9 T experimental data with the temperature, where $C_{mag}\propto T$ (solid lines are linear extrapolations). The other four panels show the temperature-dependent magnetic specific heat curves (per mole of Ce atoms) of all six samples together in a given magnetic field ((**c**) B= 0, (**d**) B= 3 T, (**e**) B= 6 T, and (**f**) B= 9 T).

Having determined the γ and α values for each sample, and in light of the fact that the magnetic contribution to the specific heat became significant only in the low-temperature limit, it is straightforward to extract $C_{mag}$ from the total specific heat C by the subtraction $C_{mag}=C-\gamma T-\alpha T^{3}$. Since γ and α are not expected to depend significantly on the external magnetic field B, this subtraction procedure is also valid for the specific heat measured in a magnetic field. The resulting magnetic specific heat of the *x* = 1.0b sample (representative of all samples) in magnetic fields of 0, 3, 6, and 9 T is presented in Figure 12a in a plot of $C_{mag}$ versus T. Here, it is important to mention that unlike the total specific heat C from Figure 11, which is presented per mole of the alloys, the magnetic specific heat $C_{mag\;}$ in Figure 12 is presented per mole of Ce atoms in the samples. The $C_{mag}\left(B,T\right)$ curves exhibit a maximum, which shifts to higher temperatures with an increasing magnetic field. Interesting behavior can be observed when the magnetic specific heat curves of the samples with different Ce concentrations are compared in the same magnetic field B. This is presented in Figure 12c–f, where the temperature-dependent magnetic specific heat curves of all six samples in a given field are shown together, with a separate panel for each field value (i.e., in Figure 12c, the $C_{mag}$ curves of all samples in the field B= 0 are shown, whereas in Figure 12d–f, the curves of all samples in the fields of 3, 6, and 9 T, respectively, are shown). A remarkable result is that the curves overlap well, forming a single $C_{mag}\left(B,T\right)$ “master” curve for each field and signifying that $C_{mag}$ (presented per mole of the Ce atoms) is the same for all samples. This demonstrates that the magnetic specific heat per single magnetic impurity (per single Ce ion) was independent of the Ce concentration $c_{imp}$ in the samples. The magnetic specific heat of the Ce*_x_*LaLuScY HEAs was hence of a single-ion character, originating either from the single-impurity Kondo effect in the entire employed range of Ce contents *x* = 0.05–1.0, from the Schottky effect, or from both.

## 4. Discussion

The presented experimental results indicate that the Kondo effect observed in the Ce*_x_*LaLuScY (*x* = 0.05–1.0) series of HEAs was of a single-impurity type for all investigated *x* values, despite the fact that the Ce concentrations were high enough to classify the alloys as dense (concentrated) Kondo systems. The single-impurity character of the Kondo effect is corroborated by the following experimental findings:

1. The prefactor $\rho_{1}$ of the Kondo term in the Hamann electrical resistivity formula (Equation (1)) was directly proportional to the Ce impurity concentration such that $\rho_{1}\propto c_{imp}$, as shown in Figure 8a and also predicted theoretically by Equation (2) for the single-impurity systems. The Kondo temperature $T_{K}$ extracted from the Hamann fits of the zero-field resistivities was, within the experimental precision, independent of the Ce concentration (Figure 8b), suggesting that the magnetic impurities were acting independently in the Kondo effect, hence supporting its single-impurity character. Here, we mention that the Kondo temperature (multiplied by the Boltzmann constant $k_{B}$) is defined as the energy scale, which limits the validity of the original Kondo’s perturbative result [9]. $T_{K}$ is lower than the temperature of the electrical resistivity minimum $T_{min}$, and the temperature range between $T_{min}$ and $T_{K}$ is considered the energy range where the exchange interaction between the localized magnetic impurity and the itinerant electrons is perturbatively weak, while at temperatures below $T_{K}$, the coupling becomes non-perturbatively strong, resulting in the formation of a Kondo-compensated singlet state of the combined impurity-itinerant electron system.
2. The magnetic susceptibility confirms that the investigated alloys were paramagnetic down to the lowest investigated temperature of 1.8 K, without any indication of collective magnetic ordering. Paramagnetism of the localized Ce moments followed the Curie–Weiss law with a relatively small (absolute) Curie–Weiss temperature $\theta_{CW}$ of about −32 K when determined from the susceptibility data at temperatures T> 50 K. The negative $\theta_{CW}$ indicates AFM interactions of the RKKY type between the Ce moments, but the interactions were too weak to induce collective magnetic ordering. The size of the Ce paramagnetic moment in this temperature range was practically identical to the theoretical value for a Ce^3+^ ion. At low temperatures, the inverse susceptibility data ${\left(\chi -\chi_{0}\right)}^{-1}$ showed a departure from the high-temperature Curie–Weiss prediction and could be analyzed by another Curie–Weiss fit that yielded a reduced (absolute) $\theta_{CW}$ and reduced effective magnetic moment $\mu_{eff}$ of the Ce ions. This is compatible with the Kondo effect, where the Ce spins are compensated by the oppositely directed spins of the conduction electrons that reduce the effective moment and consequently the strength of the RKKY interaction between the Ce moments. The compensation was not complete even at the lowest temperature T= 1.8 K (i.e., $\mu_{eff}\neq$ 0 at T→ 0), which can be attributed partially to the effect of the external magnetic field in which the experiments were conducted. (The Zeeman interaction can turn over some of the conduction-electron spins coupled by AFM interactions to the Ce spin.) It is also theoretically not a general result that the impurity moment must be completely compensated in the ground state when orbital angular momentum and crystal field terms are included in the single-impurity Kondo models [9]. However, the crystal field can also reduce the magnetic moment at low temperatures, and since the CF energy level scheme for the Ce*_x_*LaLuScY HEAs is not known yet, it is not possible to claim unambiguously the sole Kondo origin of the low-temperature deviation from the high-temperature Curie–Weiss behavior.
3. The Pauli paramagnetic spin susceptibility of the conduction electrons $\chi_{0}$ scaled linearly with $c_{imp}$ (Figure 10). Such behavior was predicted theoretically by the Kondo models that considered the magnetic impurities as independent. For example, within the non-interacting Anderson model (one of the simplest models that treats magnetic impurities in metals, originally derived for spin S= 1/2) [9,26], the electronic DOS is written as $g\left(\varepsilon \right)=g_{0}\left(\varepsilon \right)+\Delta g_{imp}\left(\varepsilon \right)$, where $g_{0}\left(\varepsilon \right)$ is the DOS of the nonmagnetic host metal, whereas $\Delta g_{imp}\left(\varepsilon \right)$ is the change in the DOS due to the magnetic impurities. The origin of the DOS change is localization of the conduction electrons for a time near the impurity due to resonant scattering at the impurity site, which induces a narrow peak in the DOS known as the virtual bound state resonance. In such a state, the conduction electrons spend a relatively larger proportion of time in the region near the impurity, but it is not a bound state because the wave functions become Bloch states far from the impurity. If the resonance occurs within the Fermi-level region, then the impurity contribution to the Pauli paramagnetic susceptibility and the electronic specific heat enhance these two physical quantities. For the noninteracting magnetic impurities, the Pauli susceptibility is written as $\chi_{P}=\chi_{P,0}+\Delta \chi_{imp}$, where $\chi_{P,0}=\mu_{0}\mu_{B}^{2}g_{0}\left(\varepsilon_{F}\right)$ refers to the nonmagnetic host, while $\Delta \chi_{imp}=\mu_{0}\mu_{B}^{2}c_{imp}\Delta g_{imp}\left(\varepsilon_{F}\right)$ is the change in the Pauli susceptibility by the magnetic impurities [9,26], depending linearly on $c_{imp}$. The linear $\chi_{0}$ versus $c_{imp}$ relation shown in Figure 10 supports the single-impurity type of Kondo effect in the Ce*_x_*LaLuScY HEAs.
4. The electronic specific heat coefficient γ increased linearly with the Ce impurity concentration such that $\gamma \propto c_{imp}$ (inset in Figure 11). Within the above discussed non-interacting Anderson model [9,26], the γ coefficient is written as $\gamma =\gamma_{0}+\Delta \gamma_{imp}$, with $\gamma_{0}=\left(\pi^{2}/3\right)k_{B}^{2}g_{0}\left(\varepsilon_{F}\right)$ describing the nonmagnetic host and $\Delta \gamma_{imp}=\left(\pi^{2}/3\right)k_{B}^{2}c_{imp}\Delta g_{imp}\left(\varepsilon_{F}\right)$ being the change introduced by the noninteracting magnetic impurities, depending linearly on $c_{imp}$. The linear γ versus $c_{imp}$ relation from Figure 11 supports the single-impurity type Kondo effect in the Ce*_x_*LaLuScY HEAs.
5. The magnetic specific heat (calculated per mole of Ce atoms) in a given (fixed) magnetic field was the same for all six samples, indicating that the magnetic specific heat per single magnetic impurity (per single Ce atom) was independent of the Ce concentration $c_{imp}$. The magnetic specific heat calculated per mole of the alloys then increased linearly with $c_{imp}$. This is in favor of the single-ion character of the magnetic specific heat, originating either from the single-impurity Kondo effect, the Schottky effect, or both. There was no sign of magnetic ordering induced by the RKKY interaction between the localized Ce moments in the magnetic specific heat down to the lowest investigated temperature.

Distinguishing between the Kondo- and Schottky-type specific heat is experimentally difficult, because both exhibit similar maximums at low temperatures. For the theoretical analysis, an appropriate Hamiltonian of the system needs to be constructed, which in the absence of a magnetic field is the sum of a single-impurity Kondo Hamiltonian pertinent to the Ce^3+^ ions with the total angular momentum quantum number J= 5/2 and the CF Hamiltonian with the crystal electric field of hexagonal symmetry that lifts the degeneracy of the six degenerate energy levels m= −5/2, −3/2,…, 5/2 into three ±m doublets. In an external magnetic field, the Zeeman Hamiltonian must be added, lifting the ±m degeneracy of the doublets to yield six nondegenerate levels. The total Hamiltonian of the problem is $H=H_{K}+H_{CF}+H_{Z}$, where $H_{K}$, $H_{CF}$, and $H_{Z}$ are the Kondo, crystal field, and Zeeman terms, respectively. The energy levels of the total Hamiltonian then need to be calculated by diagonalization, which is a complicated task due to the many-body nature of $H_{K}$. There exist various single-impurity Kondo models that treat an isolated magnetic impurity coupled antiferromagnetically by an exchange interaction to the conduction electrons of the host metal. The models most appropriate for the Ce and Yb ions are N-fold degenerate models (with N= 6 for Ce and N= 8 for Yb) like the Coqblin-Schrieffer model [9,27] (a generalization of the s-d exchange model to degenerate states) and the N-fold degenerate Anderson model with U=∞, where U denotes the strength of the Coulombic interaction [9,26]. These Kondo models have been successfully diagonalized by the Fermi liquid and Bethe ansatz approaches, combined with the 1/N expansion and large N methods (a great achievement of computational many-body physics), but these are still oversimplified for most experimental physical situations because the effects of the crystal field and the external magnetic field are not included. Theoretical approaches with the crystal field of cubic symmetry included in the calculations exist in the literature [28,29], and the case of a Ce^3+^ ion in a noncubic crystal field was also treated [30] but under the restriction that only the two low-lying doublets were taken into account (hence reducing the N= 6 case to N= 4). To the best of the authors’ knowledge, there are no reports in the literature on the treatment of a hexagonal crystal field pertinent to the Ce*_x_*LaLuScY HEAs. Finding the energy levels of the Hamiltonian $H=H_{K}+H_{CF}+H_{Z}$ by diagonalization to perform theoretical analysis of $C_{mag}$ in order to distinguish between the Kondo and Schottky effects is beyond our current capabilities (and the scope of this paper). 

The specific heat experiments presented above are, however, in favor of the Kondo origin of the magnetic specific heat. The first indication is the temperature range of $C_{mag.\;}$. The Kondo specific heat is generally nonzero in the temperature (or energy) range below the Kondo temperature $T_{K}$, where the exchange coupling between the localized magnetic impurity and the spins of itinerant electrons is non-perturbatively strong, resulting in the formation of the Kondo-compensated many-body ground state. During the formation of this state, the exchange energy of the combined impurity-itinerant electron system decreased, resulting in an energy release from the system as heat and forming an exothermic peak in the magnetic specific heat. The theoretical result obtained for the s-d exchange model with spin S= 1/2 impurities [31] (not directly applicable to the Ce*_x_*LaLuScY HEAs) predicted a peak at the temperature $T_{K}/3$. The calculations extended to impurities with higher spins S= 1 and 3/2 also predicted a peak in the magnetic specific heat at a fraction of $T_{K}$, but the magnitude of the peak decreased quite strongly with the increasing spin [32]. The experimental $C_{mag}\left(B,T\right)$ curves presented in Figure 12 reveal that the zero-field magnetic specific heat of all Ce*_x_*LaLuScY alloys was nonzero below about 12 K, with the peak being at about 3 K. This temperature range is compatible with the Kondo specific heat, since the zero-field Kondo temperatures of the Ce*_x_*LaLuScY HEAs with different Ce concentrations are all in the range $T_{K}\approx$ 12–14 K.

The second indication is the asymptotic behavior of $C_{mag}$ in the T→ 0 limit. A common result of the theoretical calculations of the Kondo specific heat using different single-impurity models is a linear temperature dependence in the low-temperature limit $T\ll T_{K}$, proceeding as $C_{mag}\propto T/T_{K}$ and being a universal function of the reduced temperature $T/T_{K}$ [25]. Vanishing of the Kondo specific heat upon T→ 0 is hence linear in terms of temperature. In contrast, vanishing of the Schottky specific heat in the T→ 0 limit is exponential [25]. The vanishing of the experimental $C_{mag}\left(B,T\right)$ curves was linear in terms of temperature, as demonstrated in Figure 12b, where the experimental data of the *x* = 1.0b sample in magnetic fields of 3, 6, and 9 T measured down to 0.35 K were extrapolated to T= 0 by linear lines. (For the zero-field magnetic specific heat, the linear extrapolation to T= 0 could not be conducted reliably because of the proximity of the maximum that made the experimental data too curved down to 0.35 K.) This result is again in favor of the Kondo origin of the magnetic specific heat.

Since the investigated Ce*_x_*LaLuScY alloys can all be classified as dense Kondo systems, (The SEM-EDS experimental Ce concentrations ranged from 1.5 to 22.5 at.%, while the dilute limit was at about 0.01 at.%.) the single-impurity character of the observed Kondo effect was surprising. To this end, it is interesting to compare the Ce*_x_*LaLuScY HEAs with other known dense Kondo systems that contain RE elements, particularly Ce, Yb, and Tm:

(i) The first kind of such systems is Kondo lattices [9], denoting chemically ordered crystalline intermetallic compounds with a regular periodic distribution of the magnetic RE elements (in abundant concentrations) on a crystal lattice. Examples include CeAl_3_, CeAl_2_, CePd_3_, RECu (RE = Ce, Yb), and TmS. In these systems, the magnetic 4*f* electrons are localized at high temperatures, and there is no significant *f*–*f* overlap. The electrical resistivity exhibits a minimum with a −lnT behavior below the minimum in much the same way as the dilute (single-impurity type) Kondo alloys. But unlike dilute Kondo alloys, instead of reaching a high constant value in the T→ 0 limit, the resistivity of a Kondo lattice passes through a maximum at a characteristic temperature $T^{*}$, called the *lattice Kondo temperature* (which is lower than the Kondo temperature $T_{K}$), below which it drops by one or two orders of magnitude. Below $T^{*}$, the RE moments become progressively compensated by the spins of conduction electrons, and at the same time, the magnetic 4*f* electrons undergo weak delocalization until complete compensation is achieved, forming a coherent state between the 4*f*-scattering sites. The electronic wave functions are now of a Bloch form, propagating in a medium in which the RE ions are arranged on a translationally periodic sublattice. The scattering of conduction electrons becomes coherent, and the lattice part of the resistivity becomes negligibly small in the T→ 0 limit. The Kondo lattices share this peculiar low-temperature behavior with heavy fermion compounds [9]. A common structural feature of the RE-containing Kondo lattices and HEAs is a periodic crystal lattice (though the lattice is topologically ordered in the former and distorted in the latter), but they differ in the chemical order on the lattice and hence in the distribution of the RE elements. While in the Kondo lattices, the RE ions are located on a translationally periodic sublattice, they are positioned randomly on the lattice sites in the chemically disordered crystalline structure of an HEA. A coherent state between the 4*f*-scattering sites cannot be achieved in an HEA, and the resistivity does not exhibit a maximum below $T_{K}$ followed by a drop at T→ 0, but it eventually saturates to a high constant value in much the same manner as it does for the single-impurity Kondo systems.
(ii) The second kind of dense Kondo system relevant for the comparison with HEAs is RE-containing magnetic metallic glasses (MGs) that exhibit the Kondo effect. A common structural property of MGs and HEAs is the random chemical disorder of the constituting elements (hence the random positioning of the 4*f*-scattering sites in the material), while they differ in the presence of a crystal lattice in the HEAs and its complete absence in the amorphous MGs. The Kondo effect in MGs is still a scarcely investigated field and has been reported thus far for the RE-containing magnetic MGs Ce*_x_*La_65−*x*_Al_10_Cu_20_Co_5_ (*x* = 10, 20, and 65 at.%) [33], (CuZr)_92.5_Al_7_RE_0.5_ (RE = Ce, Sm), Yb_12.5_Ca_50_Zn_20_Mg_17.5_, Yb_62.5_Zn_15_Mg_17.5_Cu_5_, and Sm_10_Y_45_Al_25_Cu_20_ [34]. A common feature observed in these MGs is a Kondo resistance minimum with ρ∝−lnT behavior below the minimum and a tendency toward saturation to a high constant value in the T→ 0 limit, but there is no indication of a maximum in the resistivity or its drop at T→ 0 like in the Kondo lattices. From this point of view, the investigated Ce*_x_*LaLuScY HEAs behave similarly to those MGs, whereas both are quite different from the Kondo lattices. This is not surprising because a regular periodic sublattice of the magnetic RE elements does not exist either in the MG or HEA structures. One specific feature predicted theoretically for the disordered Kondo systems is the disorder-induced distribution of Kondo temperatures $T_{K}$ [35,36], which has not been confirmed experimentally for the Kondo MGs as of yet. The same effect might also exist in HEAs, but the resistivity of the investigated Ce*_x_*LaLuScY HEAs could be well fitted within the Hamann model by using a single $T_{K}$ value without invoking the idea of its distribution.
(iii) The third kind of dense Kondo system to compare with the HEAs is the RE-containing crystalline intermetallic compounds of the chemical formula RE1*_x_*RE2_1−*x*_*M*, where RE1 is a magnetic rare earth element (mostly Ce and Yb) that is randomly substituted by a nonmagnetic rare earth element RE2 and *M* is a simple metal. Such systems can be loosely designated as chemically (substitutionally) disordered Kondo lattices, but the analogy with the true Kondo lattices is not particularly felicitous because the magnetic RE1 ions in the former systems are randomly distributed over the rare earth sites on the crystal lattice and do not form a translationally periodic sublattice like they do in the true Kondo lattices. A particularly intriguing feature of these systems is that the interactions between the RE1 magnetic moments were experimentally found to be notably reduced compared with the “normal” magnetic RE compounds, and the systems remained paramagnetic down to the lowest temperatures. Such systems also show the Kondo effect, which can astonishingly be described within the single-impurity picture, despite the concentrated amounts of the magnetic RE1 ions. Examples include the Yb*_x_*Y_1−*x*_CuAl system, which shows single-impurity Kondo behavior for *x* ≤ 0.2 [37], Ce*_x_*La_1−*x*_Cu_6_ with the single-impurity behavior for 0 < *x* < 0.7 [38], and Ce*_x_*La_1−*x*_Pb_3_, where the single-impurity behavior was observed even at a Ce content as high as *x* ≅ 0.8 [39]. The transition from the dense limit to the single-impurity limit in disordered Kondo lattices by varying the concentration of the RE1 element was also studied theoretically [40,41], but the microscopic picture of the phenomenon needs further attention. The substitutionally disordered Kondo lattices and the investigated Ce*_x_*LaLuScY HEAs appear to be closest to each other regarding the appearance of the Kondo effect. In both systems, the interactions between the magnetic RE ions are greatly reduced down to the lowest investigated temperatures such that the Kondo effect is of a single-impurity type, despite the concentrated amounts of the RE atoms. From the structural point of view, there are two common features of the disordered Kondo lattices and the investigated HEAs. The first is the presence of a crystal lattice, which is topologically distorted in both cases due to random substitution of the RE elements with slightly different atomic radii. Secondly, the magnetic RE ions are distributed randomly over the crystallographic RE sites in both systems, with none of them forming a translationally periodic magnetic 4*f* sublattice. The difference between the two systems is the size of the entropy of mixing $\Delta S_{mix}$. While in the Ce*_x_*LaLuScY, mixing of five elements makes $\Delta S_{mix}$ large enough to classify the alloys as HEAs, only two elements are randomly mixed on a particular crystallographic site in the substitutionally disordered Kondo lattices such that $\Delta S_{mix}$ does not reach a high enough value to classify the compounds as HEAs (instead, they can be classified as low-entropy alloys). In light of the similarity of the Kondo effect in these two kinds of chemically disordered crystalline systems, the difference in magnitude of the mixing entropy does not seem to play a significant role in the physics of the Kondo problem.

The reason for the strong reduction in the RKKY coupling in RE-containing HEAs and disordered Kondo lattices could be identical, originating from the chemical and topological disorder. RKKY is a long-range interaction that involves many shells of interacting neighbors, where the sign of the exchange coupling constant oscillates in space between positive and negative values on the nanometric scale. In a disordered system with random positioning of the magnetic ions on the lattice, such an alternating-sign long-range interaction may average out to a quite small value.

## 5. Conclusions

In a search for the electronic phenomena in HEAs that go beyond the independent electron description, we synthesized a series of hexagonal RE-based HEAs with the nominal formula Ce*_x_*LaLuScY by systematically varying the Ce content in the range *x* = 0.05–1.0. The measurements of electrical resistivity, magnetic susceptibility, and specific heat revealed that the Ce*_x_*LaLuScY HEAs showed the Kondo effect, which was of a single-impurity type in the entire range of employed Ce concentrations, despite the fact that the alloys were classified as dense Kondo systems. The crystal-glass duality of HEAs did not bring conceptually new features to the Kondo effect that would not be already present in other disordered (and dense) Kondo systems. The reason why the RKKY interaction between the Ce magnetic moments in the Ce*_x_*LaLuScY HEAs was reduced to the extent that the Kondo effect appeared to be of a single-impurity type despite the concentrated amounts of Ce ions is not obvious and deserves further attention, but the same phenomenon was also found in several other Ce-containing chemically disordered Kondo lattices and MGs, where the effect is also not completely understood.

While the Kondo effect is clearly a many-body physical phenomenon, its simultaneous classification as a correlated electron phenomenon needs further comments. The original Kondo’s perturbative model that is valid at temperatures $T>T_{K}$ describes a single localized quantum impurity coupled to a continuous band of delocalized and *noninteracting* electrons by an AFM exchange interaction. This “high-temperature” regime of the Kondo problem (characterized by a ρ∝−lnT dependence of the resistivity) is hence a many-body phenomenon, but it is not a correlated electron phenomenon. At sufficiently low temperatures, the normal state of most metals and alloys can be properly described only when the electron–electron (repulsive) Coulomb interaction is taken into account. The low-temperature resistivity is then dominated by electron–electron scattering in combination with *umklapp* scattering, and the Fermi liquid model of correlated electrons is able to explain the temperature dependence of the resistivity that varies as $\rho \propto T^{2}$. Metallic RE-containing alloys with partially filled *f* orbitals are described well at low temperatures as Fermi liquids, and it is reasonable to assume that the investigated RE-based Ce*_x_*LaLuScY HEAs also belong to the class of Fermi liquids. This is supported by their electrical resistivity, which is described well by the Hamann formula (Equation (1)) that contains the Fermi liquid “fingerprint” in the $qT^{2}$ term. In addition, the solutions for the Kondo models that are most appropriate for the Ce and Yb ions to describe the Kondo effect in the low-temperature (strong-coupling) regime at $T<T_{K}$ (the Coqblin–Schrieffer model and the N-fold degenerate Anderson model with U=∞) are based on the Fermi liquid theory. The Kondo effect at $T<T_{K}$ in the Ce*_x_*LaLuScY HEAs is thus a correlated electron phenomenon. In light of this, the presented study broadens the classification of HEAs to correlated–electron systems. 

Finally, it needs to be mentioned that a resistance minimum has already been reported for an HEA with the composition Al_2.08_CoCrFeNi, which is described as “Kondo-like” [42]. However, the question of whether the minimum indeed signifies the Kondo effect needs further attention, because this HEA is ferromagnetic with a relatively high Curie temperature of 870 K, and in bulk metallic alloys, the ferromagnetism and the Kondo state compete with each other. (In contrast, the two phenomena can be present simultaneously in quantum dots [43].) Apart from the zero-field electrical resistivity, there are also no complementary experiments (resistivity in the magnetic field, magnetic susceptibility, or specific heat) presented in [42] that could elucidate the situation.

## 6. Methods

The alloys were prepared by arc melting the mixtures of metals with purities of 99.99% in a Ti-gettered high-purity argon atmosphere.

XRD diffraction patterns were collected in reflection mode using a PANalytical X’Pert PRO MPD X-ray powder diffractometer (Malvern Panalytical, Almelo, The Netherlands) equipped with a primary Ge monochromator, delivering pure Cu K*α*_1_ radiation (λ= 1.54056 Å), and an X’Celerator position-sensitive detector.

SEM BSE and EDS analyses were performed by treating the samples in a high vacuum during the whole process, using the FEI FIB Helios NanoLab 650 dual-beam system (FEI, Hillsboro, OR, USA). The surfaces of the samples were cut and polished by gallium ions. SEM BSE imaging was carried out by a four-quandrant BS detector, whereas SEM-EDS chemical composition determination and elemental mapping were performed by the EDXS system from Oxford instruments with an X-max SDD detector.

HAADF-STEM micrographs and STEM-EDS elemental maps were obtained by a Cs-corrected Jeol ARM 200 CF STEM microscope (JEOL, Tokyo, Japan) equipped with an SDD Jeol Centurio energy-dispersive X-ray spectrometer. The operating voltage was set to 200 kV. The lamellae for the STEM investigations were prepared with the FEI FIB Helios NanoLab 650 dual-beam system using gallium ions.

Electrical resistivity, magnetoresistance, and specific heat measurements were performed on a PPMS 9T Quantum Design Physical Property Measurement System (Quantum Design, San Diego, CA, USA). For the resistivity measurements, rectangular bar samples of the dimensions 1 × 1 × 8 mm^3^ were used, with the long dimension oriented along the magnetic field direction to minimize the demagnetization effects. The specific heat was measured on a 2 × 2 × 0.5 mm^3^ block. Low temperatures down to 350 mK were reached by a ^3^He cryostat.

Magnetic measurements were conducted on a Quantum Design MPMS3 SQUID magnetometer equipped with a 7 T magnet and operating at temperatures between 1.8 and 400 K. The samples for the measurements were needle-shaped, with a long dimension of 4 mm and a perpendicular dimension of 0.5 mm. The long dimension was set in the direction of the external magnetic field to minimize the demagnetization effects.

## Figures and Tables

**Table 1:** Binary mixing enthalpies (in kJmol^−1^) for unlike atomic pairs constituting the Ce*_x_*LaLuScY alloys [10].

Ce	0	1	2	0
0	La	1	2	0
1	1	Lu	0	0
2	2	0	Sc	1
0	0	0	1	Y

**Table 2:** Atomic radii of the elements constituting the Ce*_x_*LaLuScY alloys (taken from [10]), together with the RT crystal structures, lattice parameters, and melting temperatures $T_{m}$ of pure metals (taken from [12]).

Element	Atomic Radius (nm)	RT Structure	Lattice Parameters (Å)	$T_{m}$ (K)
Ce	0.183	ccp dhcp	a= 5.143 a= 3.681, c= 11.857 (c/2= 5.929)	1068
La	0.188	dhcp	a= 3.770, c= 12.028 (c/2= 6.014)	1193
Lu	0.173	hcp	a= 3.511, c= 5.572	1925
Sc	0.165	hcp	a= 3.309, c= 5.268	1814
Y	0.182	hcp	a= 3.647, c= 5.728	1799

**Table 3:** List of the investigated Ce*_x_*LaLuScY samples, their nominal and SEM-EDS chemical compositions, structure, crystallographic parameters, and mass fractions of the constituent phases. The column $c_{imp}$ gives the experimental SEM-EDS concentrations of the Ce magnetic “impurities” in the samples. The accuracy in the lattice parameters is about ±1 or ±2 on the third decimal, while the accuracy in the phase fractions is up to ±5 wt.%.

Sample (Nominal Composition)	Sample (Nominal Composition in at.%)	SEM-EDS Composition (at.%)	$c_{imp}$ (at.%)	Structure	Phase Fraction (wt.%)
Ce_0.05_LaLuScY	Ce_1.2_La_24.7_Lu_24.7_Sc_24.7_Y_24.7_	Ce_1.5_La_31.0_Lu_31.0_Sc_17.1_Y_19.4_	1.5	hcp-S, a= 3.582 Å, c= 5.650 Å	37
				hcp-L, a= 3.601 Å, c= 5.683 Å	63
Ce_0.1_LaLuScY	Ce_2.4_La_24.4_Lu_24.4_Sc_24.4_Y_24.4_	Ce_2.4_La_25.9_Lu_34.6_Sc_17.3_Y_19.8_	2.4	hcp-S, a= 3.583 Å, c= 5.654 Å	40
				hcp-L, a= 3.600 Å, c= 5.686 Å	60
Ce_0.2_LaLuScY	Ce_4.8_La_23.8_Lu_23.8_Sc_23.8_Y_23.8_	Ce_3.1_La_26.6_Lu_25.8_Sc_24.6_Y_19.9_	3.1	hcp-S, a= 3.586 Å, c= 5.657 Å	37
				hcp-L, a= 3.605 Å, c= 5.688 Å	63
Ce_0.5_LaLuScY	Ce_11.2_La_22.2_Lu_22.2_Sc_22.2_Y_22.2_	Ce_10.5_La_25.2_Lu_24.5_Sc_22.7_Y_17.1_	10.5	hcp-S, a= 3.594 Å, c= 5.672 Å	30
				hcp-L, a= 3.617 Å, c= 5.715 Å	68
				ccp, a= 5.122 Å	2
Ce_1.0_LaLuScY (sample *x* = 1.0a)	Ce_20_La_20_Lu_20_Sc_20_Y_20_	Ce_22.5_La_23.3_Lu_25.8_Sc_13.4_Y_15.0_	22.5	hcp-L, a= 3.620 Å, c= 5.726 Å	97
				ccp, a= 5.176 Å	3
Ce_1.0_LaLuScY (sample *x* = 1.0b)	Ce_20_La_20_Lu_20_Sc_20_Y_20_	Ce_22.3_La_24.7_Lu_23.8_Sc_13.5_Y_15.7_	22.3	hcp-L, a= 3.620 Å, c= 5.726 Å	96
				ccp, a= 5.180 Å	4

## Data Availability

The data presented in this study are available on request from the corresponding authors.

## Supplementary Materials

The following supporting information can be downloaded at https://www.mdpi.com/article/10.3390/ma16247575/s1. S-I. Feasibility of producing solid solution phases in the Ce*_x_*LaLuScY (*x* = 0.05–1.0) system and their conformation to the definition of a high-entropy alloy; S-II. SEM-EDS elemental maps of the Ce*_x_*LaLuScY samples *x* = 0.05, *x* = 0.1, *x* = 0.2, *x* = 1.0a, and *x* = 1.0b; S-III. STEM-EDS analysis of the *x* = 0.2 sample; S-IV. Electrical resistivity: raw data; S-V. Magnetic field dependence of the Kondo resistivity fit parameters; S-VI. Analysis of magnetic susceptibility of the FM-contaminated samples.
