# Supplementary material for: The Kondo Effect in CexLaLuScY (x = 0.05–1.0) High-Entropy Alloys
Source: Materials (Basel). 2023 Dec 9;16(24):7575. doi: 10.3390/ma16247575 (PMC10744949; doi:10.3390/ma16247575)
Supplement: Supplementary file 1 [file materials-16-07575-s001.zip › materials-2752022-supplementary.pdf]

Supplementary Material

Kondo effect in the $Ce_xLaLuScY$ (x = 0.05–1.0) high-entropy alloys

Julia Petrović [1], Stanislav Vrtnik [1], Andreja Jelen [1], Primož Koželj [1,2], Jože Luzar [1], Peter Mihor [1], Qiang Hu [3,*], Magdalena Wencka [1,4], Bojan Ambrožič [5], Anton Meden [6], Goran Dražić [7], Sheng Guo [8], Janez Dolinšek [1,2,*]

[1] Jožef Stefan Institute, Jamova 39, SI-1000 Ljubljana, Slovenia

[2] University of Ljubljana, Faculty of Mathematics and Physics, Jadranska 19, SI-1000 Ljubljana, Slovenia

[3] Institute of Applied Physics, Jiangxi Academy of Sciences, Changdong Road 7777, Nanchang 330096, PR China

[4] Institute of Molecular Physics, Polish Academy of Sciences, Smoluchowskiego 17, PL-60-179 Poznań, Poland

[5] Center of Excellence in Nanoscience and Nanotechnology, Jamova 39, SI-1000 Ljubljana, Slovenia

[6] University of Ljubljana, Faculty of Chemistry and Chemical Technology, Večna pot 113, SI-1000 Ljubljana, Slovenia

[7] National Institute of Chemistry, Department of Materials Chemistry, Hajdrihova 19, SI-1000 Ljubljana, Slovenia

[8] Industrial and Materials Science, Chalmers University of Technology, SE-41296 Göteborg, Sweden

* Correspondence: q-fei618@qq.com (Q.H.); janez.dolinsek@ijs.si (J.D.)

S-I. FEASIBILITY OF PRODUCING SOLID SOLUTION PHASES IN THE Ce_xLaLuScY (x = 0.05–1.0) SYSTEM AND THEIR CONFORMATION TO THE DEFINITION OF A HIGH-ENTROPY ALLOY.

The feasibility of producing solid solution phases in the Ce_xLaLuScY (x = 0.05–1.0) system was assessed theoretically via the commonly used empirical criteria to predict solid solution formation in multicomponent alloys [1]. The criteria rely on the Gibbs free energy of mixing, $\Delta G_{mix} = \Delta H_{mix} - T\Delta S_{mix}$, where ΔH_{mix} and ΔS_{mix} are the mixing enthalpy and mixing entropy of the alloy, respectively. The mixing enthalpy is calculated from $\Delta H_{mix} = 4\sum_{i,j=1,j>i}^{n} c_i c_j \Delta H_{mix}^{ij}$, where c_i is the concentration of component i and n is the number of components in the alloy ($n = 5$ in this case). The mixing entropy is calculated from $\Delta S_{mix} = -R\sum_{i=1}^{n} c_i \ln c_i$, where R is the gas constant. Convenient assessment parameters are the thermodynamic parameter $\Omega = \bar{T}_m \Delta S_{mix}/|\Delta H_{mix}|$, where $\bar{T}_m = \sum_i c_i T_m^i$ is the composition-averaged melting temperature (T_m^i is the melting temperature of the component i) and the atomic-size-difference (geometric) parameter $\delta = 100(\sum_{i=1}^{n} c_i(1 - r_i/\bar{r})^2)^{1/2}$, where $\bar{r} = \sum_i c_i r_i$ is the composition-averaged atomic radius (r_i is the atomic radius of the component i). For the elemental concentrations c_i in the alloys, nominal concentrations in at.% were taken (e.g., the composition of the alloy $Ce_{0.05}$LaLuScY recalculated in at.% is $Ce_{1.2}La_{24.7}Lu_{24.7}Sc_{24.7}Y_{24.7}$). The so determined values of the quantities ΔH_{mix}, ΔS_{mix}, Ω, and δ (together with $\bar{r}$ and $\bar{T}_m$) of all five alloys are collected in Table S1. The values fall in the ranges $1.01 \leq \Delta H_{mix} \leq 1.12$ kJmol^{-1}, $11.93 \leq \Delta S_{mix} \leq 13.38$ JK^{-1}mol^{-1}, $18.64 \leq \Omega \leq 19.92$ and $4.59 \leq \delta \leq 4.92$%. The large Ω values are a consequence of the very small ΔH_{mix} values, which are in the denominator of the expression for this parameter. Though there are no universal criteria for the formation of a solid solution phase, the approach by Zhang *et al*. [2] states that simple solid

solutions form when $\Omega \geq 1.1$ and $\delta \leq 6.6\%$. In the approach by Guo *et al.* [3], it is stated that the parameters ΔH_{mix} and δ should be used together to identify the formation of a solid solution, which forms when the two parameters simultaneously satisfy the conditions $-11.6 \leq \Delta H_{mix} \leq 3.2$ kJmol^{-1} and $0 \leq \delta \leq 6.6\%$. The parameter values collected in Table S1 satisfy well both types of criteria for all five $Ce_xLaLuScY$ alloys, so that the formation of a solid solution phase is highly probable.

Table S1. Composition-averaged atomic radius $\bar{r}$ and melting temperature $\bar{T}_m$, mixing entropy ΔS_{mix}, mixing enthalpy ΔH_{mix}, thermodynamic parameter Ω and atomic-size-difference (geometric) parameter δ of the $Ce_xLaLuScY$ (x = 0.05, 0.1, 0.2, 0.5, and 1.0) alloys.

x	$\bar{r}$ (nm)	$\bar{T}_m$ (K)	ΔS_{mix} (JK^{-1}mol^{-1})	ΔH_{mix} (kJmol^{-1})	Ω	δ (%)
0.05	0.177	1675	11.93	1.01	19.75	4.92
0.1	0.177	1668	12.19	1.02	19.88	4.91
0.2	0.177	1653	12.57	1.04	19.92	4.87
0.5	0.178	1614	13.15	1.09	19.53	4.76
1.0	0.178	1560	13.38	1.12	18.64	4.59

Regarding the question whether the solid solution phases expected to form in the $Ce_xLaLuScY$ (x = 0.05–1.0) system can indeed be termed as HEAs, we adopt the criterion by Yeh [4], who has divided multicomponent alloys according to the magnitude of their mixing entropy into low-entropy alloys ($\Delta S_{mix} \leq R$), medium-entropy alloys ($R < \Delta S_{mix} < 1.5R$) and high-

entropy alloys ($\Delta S_{mix} \geq 1.5R$). By rewriting the ΔS_{mix} values from Table S1 into multiples of R, we find that the alloys x = 0.05 ($\Delta S_{mix} = 1.44R$) and x = 0.1 ($\Delta S_{mix} = 1.47R$) formally belong to the medium-entropy alloys, while the alloys x = 0.2 ($\Delta S_{mix} = 1.51R$), x = 0.5 ($\Delta S_{mix} = 1.58R$) and x = 1.0 ($\Delta S_{mix} = 1.61R$) conform to the definition of a high-entropy alloy. In the main paper, we are nevertheless denoting all five alloys by the generic name HEA.

S-II. SEM-EDS ELEMENTAL MAPS OF THE Ce_xLaLuScY SAMPLES *x* = 0.05, *x* = 0.1, *x* = 0.2, *x* = 1.0a and *x* = 1.0b.

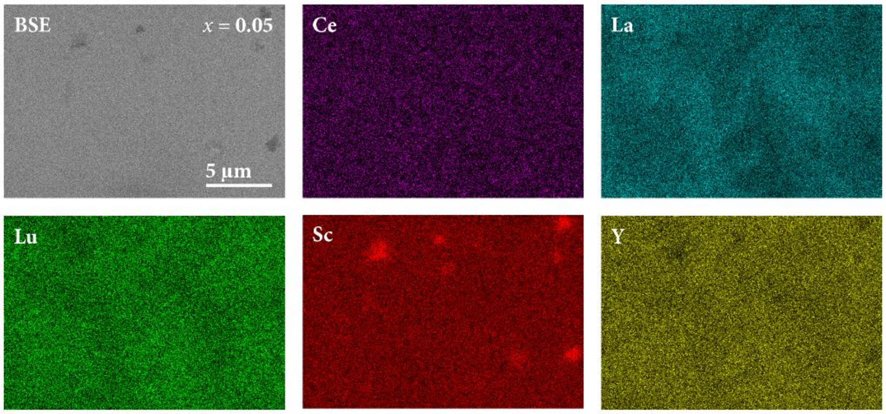

Figure S1. SEM-EDS elemental maps of the x = 0.05 sample.

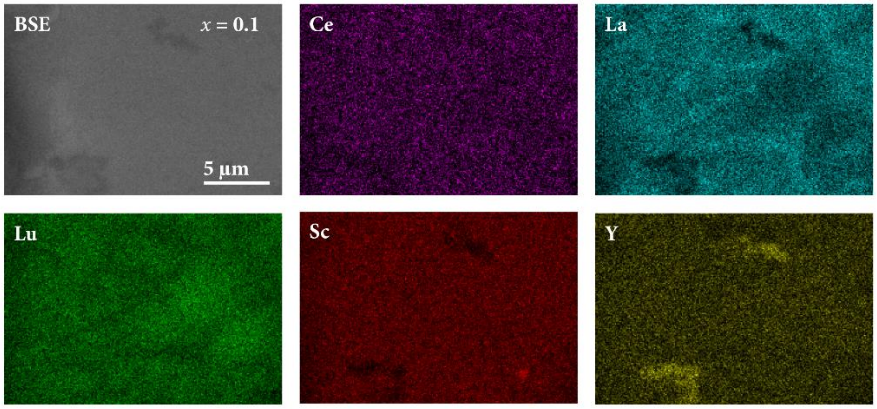

Figure S2. SEM-EDS elemental maps of the x = 0.1 sample.

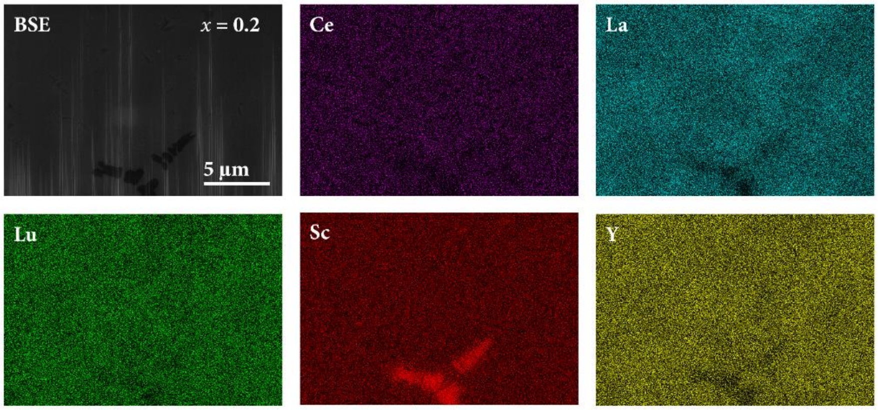

Figure S3. SEM-EDS elemental maps of the $x = 0.2$ sample.

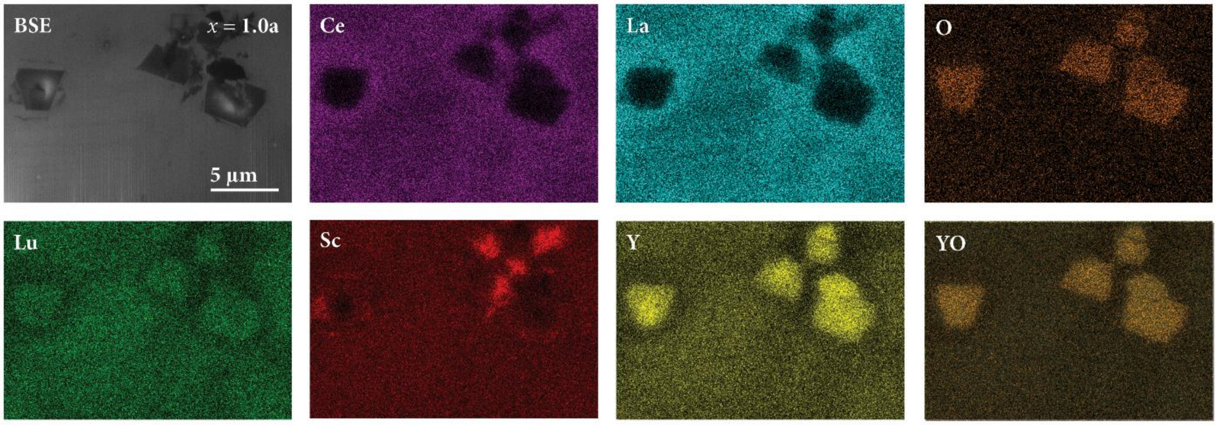

Figure S4. SEM-EDS elemental maps of the $x = 1.0a$ sample. YO is a sum of the maps Y and O.

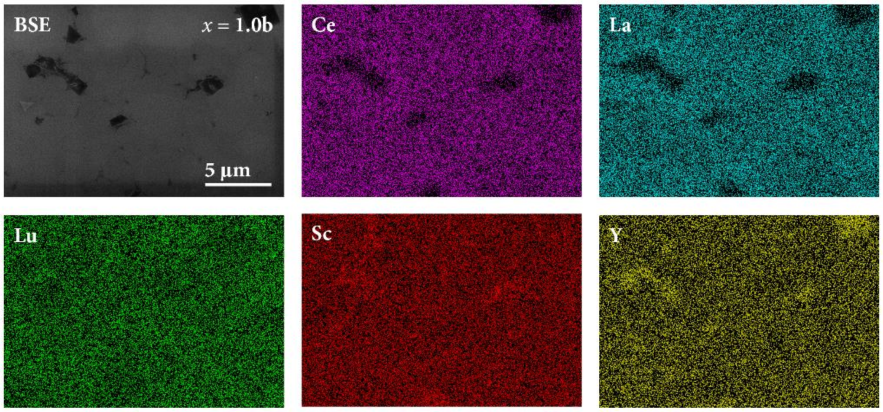

Figure S5. SEM-EDS elemental maps of the $x = 1.0b$ sample.

S-III. STEM-EDS ANALYSIS OF THE x = 0.2 SAMPLE.

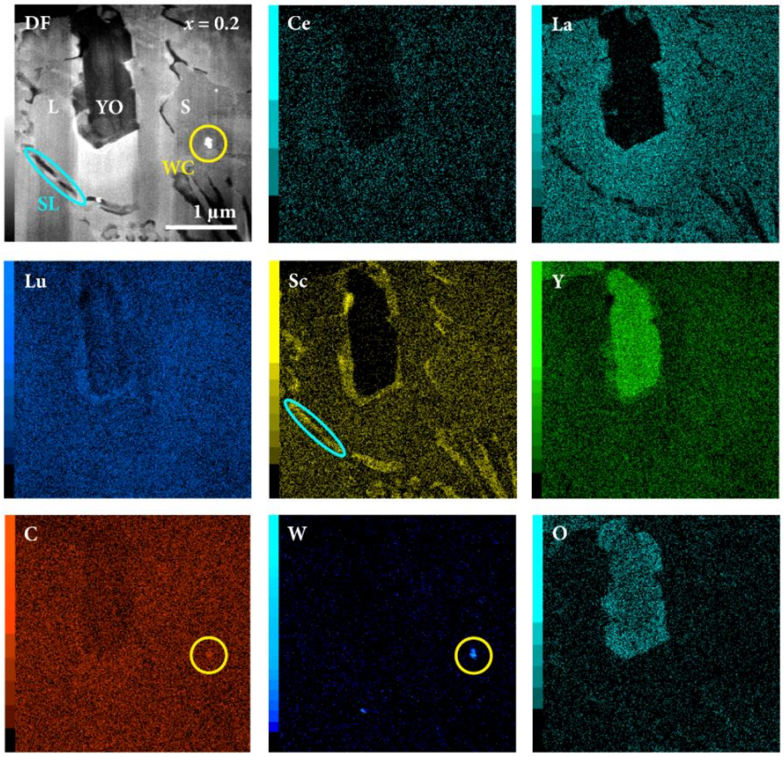

Figure S6. STEM-EDS elemental maps of the x = 0.2 sample. Upper left panel shows the STEM-DF image, where the character S denotes the region of the hcp-S phase, L denotes the hcp-L phase, SL denotes the Sc,Lu-rich defects (one is encircled light blue), YO is the yttrium-oxide region, whereas WC marks the tungsten carbide particle (encircled yellow). Other panels are the STEM-EDS elemental maps (the maps of oxygen, tungsten and carbon are also included).

Figure S6 shows the STEM-EDS analysis of the x = 0.2 sample, by examining the surface area of an approximate size 3.5×3.5 µm^2. The STEM-DF (dark field) image is presented in the upper left panel, where the character S denotes the region of the hcp-S phase, L denotes the hcp-L phase, SL

denotes the Sc,Lu-rich defects (one is encircled light blue), YO is the yttrium-oxide region, whereas WC marks the tungsten carbide particle (encircled yellow). Other panels of Figure S6 are the STEM-EDS elemental maps (the maps of oxygen, tungsten and carbon are also included). Away from the defects, the five elements Ce, La, Lu, Sc and Y are quite homogeneously dispersed, confirming random mixing of the elements within the hcp matrix on the sub-micrometer scale. The STEM-EDS point analysis yielded the chemical composition of the hcp-S phase as $Ce_{2.3}La_{23.6}Lu_{26.0}Sc_{27.0}Y_{21.1}$, with the sum of concentrations of the two smaller elements Sc + Lu amounting to 53 at.%, while the sum of the three bigger elements Ce + La + Y is 47%. The composition of the hcp-L phase was determined as $Ce_{4.2}La_{34.0}Lu_{19.0}Sc_{16.0}Y_{26.8}$, with the sums Sc + Lu of 35% and Ce + La + Y of 65%. These STEM-EDS compositions are in fair agreement with the SEM-EDS (average) composition over the two hcp phases $Ce_{3.1}La_{26.6}Lu_{25.8}Sc_{24.6}Y_{19.9}$ given in Table 3 of the main paper.

S-IV. ELECTRICAL RESISTIVITY – RAW DATA.

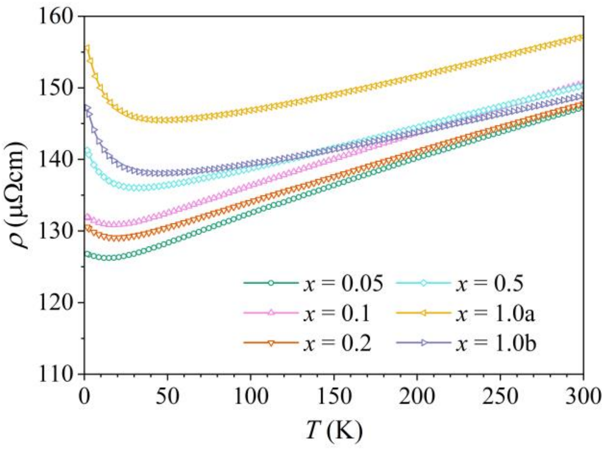

Figure S7. Temperature-dependent electrical resistivity $\rho(T)$ of the Ce_xLaLuScY samples in zero magnetic field.

S-V. MAGNETIC-FIELD DEPENDENCE OF THE KONDO-RESISTIVITY FIT PARAMETERS.

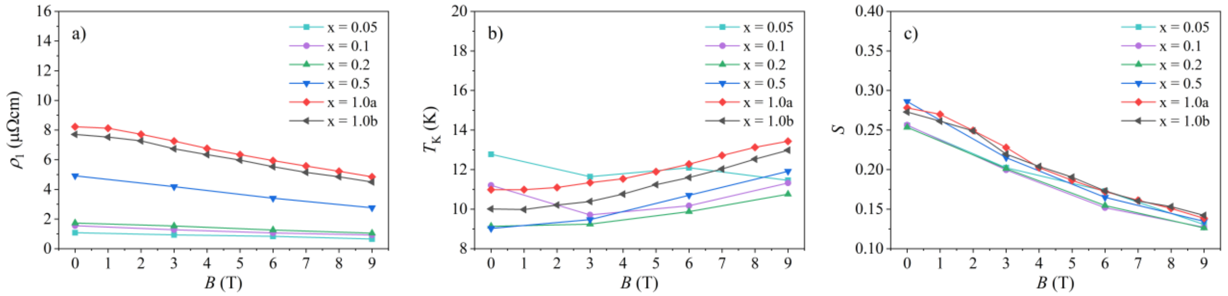

Figure S8. Dependence of the fit parameters ρ_1, T_K and S of the Kondo term in the Hamann resistivity formula on the magnetic field B for the six investigated Ce$_x$LaLuScY samples (lines connect the points).

S-VI. ANALYSIS OF MAGNETIC SUSCEPTIBILITY OF THE FM-CONTAMINATED SAMPLES.

The tiny extrinsic FM contamination that was present in several Ce$_x$LaLuScY samples has manifested as hysteresis in the magnetization versus the magnetic field, $M(H)$, curves. In Figure S9, the $M(H)$ curves of the x = 0.1 non-contaminated sample and the x = 0.5 FM-contaminated sample are compared at temperatures 1.8 and 300 K. While the non-contaminated sample does not exhibit hysteresis, hysteretic behavior is observed for the contaminated sample. The hysteresis loops close up in a field of $\mu_0 H \approx 0.3$ T, which is a typical value for FM-type loops. The extrinsic origin of the contamination is corroborated by (1) the fact that Ce does not show ferromagnetism,

while other elements in the $Ce_xLaLuScY$ HEAs are nonmagnetic and (2) the persistence of the hysteresis also at room temperature (300 K).

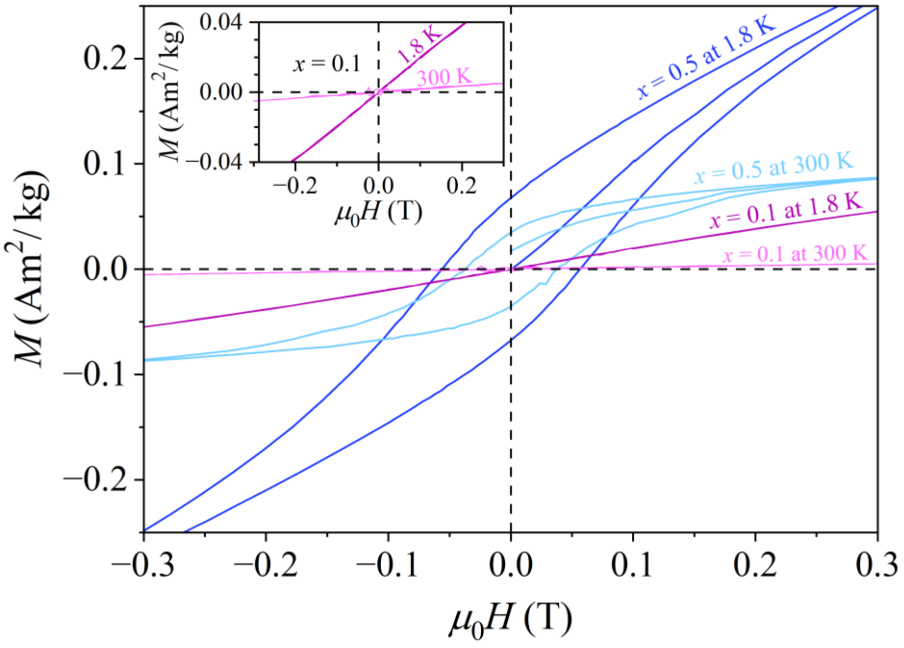

Figure S9. $M(H)$ curves of the x = 0.1 non-contaminated sample and x = 0.5 FM-contaminated sample at temperatures 1.8 and 300 K. The curves were determined in the field range ±7 T, but only the expanded portion in the range ±0.3 T around the origin is shown to demonstrate the hysteresis for the x = 0.5 sample and its absence for the x = 0.1 sample. The two $M(H)$ curves of the x = 0.1 non-contaminated sample are also shown on an expanded vertical scale in the inset. Since the FM contamination is of extrinsic origin, the magnetization is given per mass of the samples.

To check for the accuracy of the "differential" method to determine the susceptibility from the magnetization difference $\Delta M = M(H_2) - M(H_1) = (\chi_0 + \chi_{CW})(H_2 - H_1)$, in order to eliminate the FM contribution in FM-contaminated samples (as discussed in the paragraph *3.2. Magnetic susceptibility* of the main paper), we have first reanalyzed the susceptibility of the x =

0.1 non-contaminated sample with this method and compared it to the result obtained on the same sample by the “direct” method, where the magnetization measured in a single field, $M = (\chi_0 + \chi_{CW})H$, was analyzed. The result of the direct method is already shown in Figure 9 of the main paper and those data will be used for comparison with the differential method (recall that the Curie-Weiss analysis in Figure 9b was done on the magnetization measured in a 5-T field). For the differential method, the magnetizations measured in the fields 3 T, 5 T and 7 T were taken (according to Figure S9, these fields are high enough that the FM magnetization of the contaminated samples is already saturated) and the $M(5\mathrm{T}) - M(3\mathrm{T})$ and $M(7\mathrm{T}) - M(3\mathrm{T})$ differences were analyzed to extract the susceptibility $\chi_0 + \chi_{CW}$. In Figure S10a, the temperature-dependent susceptibilities $\chi = M/H$ obtained from the $M(5\mathrm{T})$ by the direct method and from the $M(5\mathrm{T}) - M(3\mathrm{T})$ and $M(7\mathrm{T}) - M(3\mathrm{T})$ using the differential method are shown on the same graph. Since the x = 0.1 sample is free of the FM contamination, the two methods are expected to give identical result. We observe that the three sets of data coincide well at temperatures above 50 K, so that the Pauli susceptibility χ_0 is accurately determined by both methods. At temperatures below 50 K, the susceptibilities start to differ, where the two susceptibilities determined by the differential method are a bit lower than the one determined by the direct method and the susceptibility obtained from $M(7\mathrm{T}) - M(3\mathrm{T})$ is additionally a bit lower than the one determined from $M(5\mathrm{T}) - M(3\mathrm{T})$. An especially disadvantageous feature of the differential method is a maximum in the susceptibility at the lowest temperatures obtained from both magnetization differences (indicated by an arrow in Figure S10a), which is obviously an artefact of the subtraction procedure. For that reason, the low-temperature analysis of the susceptibility by the differential method is inadequate.

The comparison of the two methods of analysis is also shown in Figure S10b, where the same three sets of data are presented in the $(\chi - \chi_0)^{-1}$ vs. T plots, hence showing only the Curie-Weiss part of the susceptibility. For the direct method, the high-temperature Curie-Weiss analysis has yielded the fit parameter values $\mu_{eff} = 2.56\mu_B$ and $\theta_{CW} = $ –32 K, to be contrasted to $\mu_{eff} = 2.58\mu_B$ and $\theta_{CW} = $ –35 K obtained by the differential method. The two sets of fit parameters agree well, so that the high-temperature Curie-Weiss analysis is equally precise for both methods. At low temperatures (below about 50 K), the experimental data obtained by the differential method deviate quite significantly from the (more reliable) data obtained by the direct method. In addition, the differential data depend slightly on the choice of the two magnetic fields (i.e., the data obtained from $M(7\text{T}) - M(3\text{T})$ are different from those obtained from $M(5\text{T}) - M(3\text{T})$), so that the low-temperature Curie-Weiss analysis by the differential method is less proper and precise. The artefact of subtraction (a minimum at the lowest temperatures in this kind of a plot) is also detrimental for the quantitative analysis of the data below 50 K.

Due to the above described inadequacy of analyzing the magnetic susceptibility of the FM-contaminated samples at low temperatures by the differential method, the evolution of the Kondo effect with the Ce impurity concentration c_{imp} (mirrored in the changes of the effective magnetic moment μ_{eff} and the Curie-Weiss temperature θ_{CW} with c_{imp} below the Kondo temperature T_K) could not be systematically investigated for the entire set of the Ce_xLaLuScY samples. The Pauli susceptibility χ_0 was, however, reliably determined by the differential method also for the FM-contaminated samples and the χ_0 versus c_{imp} relation for all samples is shown in Figure 10 of the main paper.

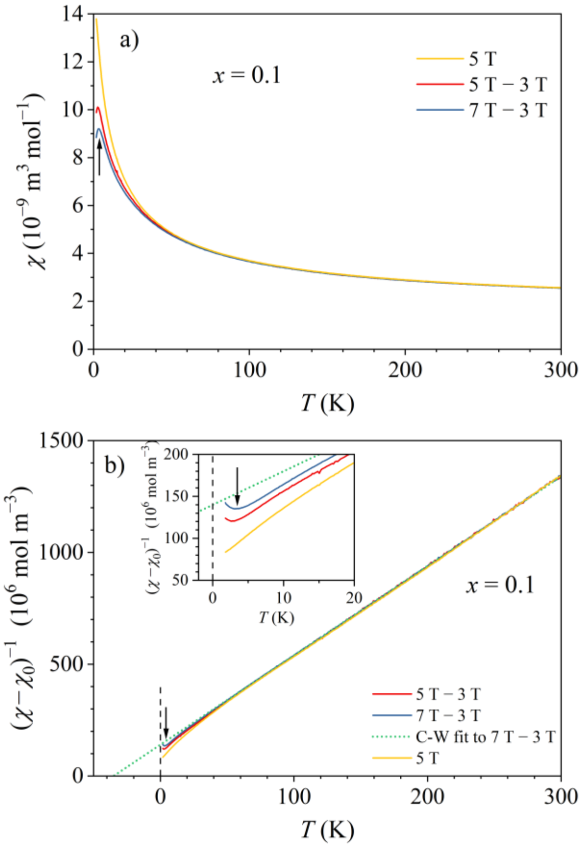

Figure S10. (a) Temperature-dependent susceptibilities $\chi = M/H$ of the $x = 0.1$ non-contaminated sample, obtained from the $M(5\mathrm{T})$ magnetization by the direct method and from the $M(5\mathrm{T}) - M(3\mathrm{T})$ and $M(7\mathrm{T}) - M(3\mathrm{T})$ magnetization differences using the differential method. The arrow denotes the maximum in the susceptibilities obtained from the magnetization differences, which is an artefact of the subtraction procedure. **(b)** The same three sets of data presented in a $(\chi - \chi_0)^{-1}$ vs. T plot that show the Curie-Weiss part of the susceptibility only. The arrow denotes the artefact of the subtraction procedure in the differential susceptibilities, which appears as a minimum in this kind of a plot. The high-temperature Curie-Weiss fit (for $T > 50$ K) to the susceptibility obtained from the $M(7\mathrm{T}) - M(3\mathrm{T})$ magnetization difference is also shown as a dotted green line (at temperatures $T > 50$ K, this line is indistinguishable from the experimental data). The low-temperature portion of all data is presented on an expanded scale in the inset.

References

1. Murthy, B.S.; Yeh, J.-W.; Ranganathan, S. *High-Entropy Alloys*; Elsevier: Amsterdam, The Netherlands, 2014.

2. Zhang, Y.; Yang, X.; Liaw, P.K. Alloy design and properties optimization of high-entropy alloys. *JOM* **2012**, *64*, 830–838.

3. Guo, S.; Hu, Q.; Ng, C.; Liu, C.T. More than entropy in high-entropy alloys: Forming solid solutions or amorphous phase. *Intermetallics* **2013**, *41*, 96–103.

4. Yeh, J.W. Alloy design strategies and future trends in high-entropy alloys. *J. Met.* **2013**, *65*, 1759–1771.
